# Two new Later Stone Age sites from the Final Pleistocene in the Falémé Valley, eastern Senegal

**DOI:** 10.1371/journal.pone.0294346

**Published:** 2024-03-28

**Authors:** Matar Ndiaye, Laurent Lespez, Chantal Tribolo, Michel Rasse, Irka Hadjas, Sarah Davidoux, Éric Huysecom, Katja Douze

**Affiliations:** 1 Department of Human Sciences, Laboratory of Prehistory and Protohistory, Institut Fondamental d’Afrique Noire (IFAN), University of Cheikh Anta Diop, Dakar, Senegal; 2 Department of Geography, Laboratory of Physical Geography (LGP), CNRS-UMR 8591, University Paris-Est Creteil, Meudon, France; 3 Department of Archaeosciences Bordeaux, University Bordeaux-Montaigne, Pessac, France; 4 Department of Geography, Maison de l’Orient et de la Méditerranée (ARCHÉORIENT), University Lumière - Lyon II, Lyon, France; 5 Laboratory of Ion Beam Physics (LIP), ETH-Zürich, Zürich, Switzerland; 6 Laboratory of Archaeology of Africa & Anthropology (ARCAN), University of Geneva, Genève, Switzerland; Sapienza University of Rome: Universita degli Studi di Roma La Sapienza, ITALY

## Abstract

The understanding of cultural dynamics at work at the end of the Final Pleistocene in West Africa suffers from a significant lack of excavated and dated sites, particularly in the Sahelian and Sudanian ecozones. While the Later Stone Age shows varied behavioral developments in different parts of the continent, the chrono-cultural framework of this period remains largely unknown in West Africa. We report on archaeological, geomorphological, and chronological research on two Final Pleistocene Later Stone Age sites in the Falémé Valley, eastern Senegal. Optically stimulated luminescence ages place the site of Toumboura I-2017 between 17 ± 1 and 16 ± 1 ka and the Ravin de Sansandé site between 13 ± 1 ka and 12 ± 1.1 ka. The excavated lithics show typical Later Stone Age industries, characterized by *chaînes opératoires* of core reduction mainly producing flakes and bladelets as well as blades and laminar flakes. Segments dominate the toolkits but a few backed bladelets and end-scrapers on flake blanks were recognized. Local raw materials were used, with a preference for chert and quartz, as well as greywacke. These Later Stone Age lithic assemblages are the oldest known in Senegal so far and add to the small number of sites known in West Africa for this period, which are mainly located farther south, in sub-tropical ecozones. The Later Stone Age sites of the Falémé Valley are contemporaneous with typical Middle Stone Age technologies in Senegal dated to at least the Pleistocene/Holocene transition. Our results thus provide new archaeological evidence highlighting the complex cultural processes at work during the Final Pleistocene in West Africa.

## 1. Introduction

The Later Stone Age (LSA) in West Africa develops in the context of important ecosystem changes during the Final Pleistocene and is only rarely discovered in stratified contexts. Tools from the Later Stone Age are more diverse, suggesting a rhythm of innovation and the emergence of distinct cultural identities. Different groups sought to produce microlithic-type tools and geometric or bifacial armatures [1, 2]. The African LSA is generally perceived as rather homogeneous in its evolution, or at least showing continuity of certain technical behaviors, as seen at sites along the Central and West Africa at Njuinye, Shum Laka in Cameroon, at Bingerville in Ivory Coast, and at Iho Eleru in Nigeria [3–12]. In the Western Central Africa the LSA emerged between 40 ka and 20 ka with a combination of typical LSA characteristics (geometric microliths including segments) and often a low presence of large tools such as wide heavy scrapers, notches, drills and denticulates. In the lower unit of layer 3 at the Njuinye site, quartz debitage has been found, which is relatively unstandardized and of microlith affiliation [7, 13, 14]. For example in Senegal, the oldest LSA sites known so far are dated back to the Final Pleistocene-Early Holocene and are contemporaneous to some of the latest MSA contexts known from the region so far [15–18]. Given the asynchrony in cultural change, thus it is difficult to generalize about the period of the emergence of the first LSA industries in West Africa [13, 19–21]. The Falémé Valley, in eastern Senegal, is the only location in the country where well-documented stratified Later Stone Age sites have been described so far [5, 6, 22], including preliminary descriptions of Toumboura I site [22] and in-depth analysis of Fatandi V site, dated to the Final Pleistocene to Holocene transition [20]. The Fatandi V site was the first stratified and dated LSA occurrence discovered in Senegal’s Falémé Valley. The knapped stone assemblage at Fatandi V clearly fits into the tradition of bladelet industries with geometric segments, as known from MIS 2 in the region [22, 23]. Recent investigations in the same valley have documented two older sites with well-contextualized and consistent microlithic assemblages, including typical Later Stone Age geometric tools [20].

This article reports the results of archaeological, geomorphological, and chronometric research conducted at these two sites, namely Toumboura I-2017 (hereafter TMBI-2017) and Ravin de Sansandé (RDS), excavated between 2017 and 2020. Toumboura I-2017 was previously described as Toumboura I, based on preliminary results from the test-excavations led on the site in 2014 [20, 22, 23]. Our study focuses on the technological and typological characterization of Later Stone Age lithic assemblages, allowing us to understand the technical dynamics at work during the Final Pleistocene. Geomorphological and chronostratigraphic analyses conducted on sedimentary deposits containing the lithic assemblages attest to the presence of a well-developed LSA in the Falémé Valley at the end of the Final Pleistocene. The archaeological data collected at both sites suggest that environmental factors played a significant role in the differentiation of cultural practices and material production. However, throughout the Final Pleistocene, we observe a juxtaposition of the contemporaneous use of MSA and LSA technologies by separate populations [20, 24, 25]. Although the MSA may be defined differently in terms of space and time from one region of the world to another, but generally it starts around 300,000 BC and finishes around 30,000 BC [26]. It is characterized above all by the diversification and standardization of artifacts, the development of bifacial technology and laminar debitage, but also by the acquisition of cultural behaviors linked to the transformation of organic materials, ritual and artistic practices and a vast network of exchanges [27, 28]. These new data give us an opportunity to outline hypotheses on the technical and adaptive behaviors that developed in the region at the end of the Pleistocene.

## 2. Material and method

### 2.1. Lithic study

For this study a total of 1701 artifacts from TMB-2017 and a total of 778 artifacts from RDS were analysed. The study began with the recognition of diagnostic blanks, tools, cores and debris. Diagnostic blanks include all complete pieces such as flakes, blades, bladelets, and laminar flakes with no obvious distal, mesial, or proximal fractures. Only complete tools and cores, again lacking obvious distal, mesial, or proximal fractures were fully analyzed for this study. Debris, defined as undeterminate pieces that do not fit into any specific technological category, were not fully analyzed. Once cleaned, each diagnostic blank and complete tool and core was numbered and recorded in a Microsoft Access database. The extent and types of analyses used were determined by the dimensions of artifacts. Artifacts with dimensions ≥30 mm and widths ≥ 10 mm were studied individually. Debris, small flakes ≤ 20 mm, very small flakes ≤ 10 mm, and fragments were counted and then removed from further study. We considered these elements to correspond to undistinguished knapping waste. Several raw material nodules that did not allow for the identification of an obvious striking platform or debitage surface were also removed from the study. It is possible that these materials were transported to the sites and abandoned without being knapped.

The study of the lithic assemblages followed the approach of reconstructing the *chaîne opératoire*, which is understood to be a dynamic process, from the acquisition of raw material to the abandonment of products [29]. The identification of descriptive attributes (ID number, raw material, dimensions, morphology, types of blanks, morpho-technological or typological categories, percentage and location of cortex, direction of removals, platform types, and distribution of retouch, among other observations) was carried out for each diagnostic blank and tool. For the cores, observations about the removal negatives, the striking platforms, and the debitage surfaces, were recorded. This allows a better understanding of the selection of materials, methods, and objectives of core reduction, particularly in terms of production of blanks and tools. Geometric pieces are considered microliths with the silhouette of a segment of a circle. They generally present an arc with abrupt retouching opposite to a sharp edge that may be straight, rough, or with partial or, rarely, complete semi-abrupt retouching [30]. For the cores, five modes of exploitation have been identified. Opportunistic cores are unprepared. In fact, a number of flakes have simply been opportunistically detached from the stone’s natural surface. Simple cores refer to the exploitation of a single prepared striking platform allowing the extraction of two to three blanks. Frontal cores are those exploited on a single facade of the nodule [31]. Semi-rotating cores allow the homogeneous and quasi-circular detachment from the nodules. Peripheral cores show discontinuous exploitation on the edges of the nodules [29, 31].

### 2.2. ^14^C and OSL dating method

Radiocarbon dating was performed on charcoal and optically stimulated luminescence (OSL) dating on soil samples.

#### 2.2.1. ^14^C dating method.

Prior to radiocarbon analysis, samples of charcoal were treated with acid and base to remove contamination with carbonates and humic acids, respectively [32, 33]. In the next step, clean and dry sample materials were weighed into aluminum boats for combustion in the elemental analyzer and a subsequent graphitization [34]. The graphite samples pressed into the aluminum cathodes were analyzed using the MICADAS system at the ETH Zurich [35, 36]. Radiocarbon ages were calculated following convention [37] and calibrated using the OxCal online program [38] and Intcal13 calibration curve [39].

#### 2.2.2. OSL dating method

Sediment samples were taken for dating by Optically Stimulated Luminescence (OSL). At RDS, samples S15 and S16 come from unit 3, at ca. 0.9 m below the surface (-1.75 m below the datum point). They were taken 14 m from each other. No direct sampling was done at TMBI-2017, but the natural exposure of the stratigraphic sequence in the reference trench named Toumboura I was sampled (see section 3). From bottom to top, T1 came from the U_J_ unit [40], T2 and T3 from the U_S_ unit, T4, T5 and T6 from the U_C_ unit, T7 and T8 from the U_G_ unit, and T9 from more recent colluviums (Fig 2). Sample T7 is located at the base of the UG unit and is dated by two OSL ages at 17 ± 1 ka and 16 ± 1 ka. This horizon corresponds to grey clayey silts with very fine grey-white sands from which the Toumboura I Later Stone Age assemblage is derived. Note that the ages for T1 to T9 presented here are those published in Lebrun and al., [41] and Lebrun [42]. However, a revision has been made by using the single grain technique for all samples instead of the previous multi-grain technique, and by improving dose rate for the artificial sources attached to the OSL readers.

The sediment samples were taken by night under subdued red-orange light, after scratching the surface exposed to daylight. In the lab, each sediment sample was homogenized and subdivided. A part (about 20 g) has been prepared for the determination of the radioisotopic content by high resolution gamma spectrometry. It was dried, finely crushed, sealed in a plastic box, and stored for at least one month. The other part (40–100 g) was prepared for the determination of the equivalent dose: the 100–140μm or 200–250μm fractions have been extracted by wet sieving. Carbonates and organic materials were eliminated with HCl (10%) followed by H O_22_ (30%). Quartz, feldspar and heavy minerals were separated with high density hetero-polytungstate of sodium at 2.72, 2.62 and 2.58 g/cm^3^. Then quartz grains were HF etched for 60 min (HF 40%, followed by HCl 10%). The grains were located on single grain discs displaying 100 holes of 150 μm or 300 μm diameter and depth, so that each hole contained only one grain.

Equivalent doses (De) were determined on single grains using the Single Aliquot and Regenerative Dose (SAR) protocol [43]. First, tests were performed in order to check that 1) the quartz sample was not contaminated by feldspar grains [44], 2) the signal was dominated by the fast component, and 3) the SAR protocol with adjusted parameters allowed a minimum to recover a known-laboratory given dose (dose recovery tests) (sup mat). Grains were selected following their sensitivity (signal> 3 x background; test dose error < 10%), their recovery (signal for a null dose <5% of the highest regenerative dose), and their saturation (though no D_0_ threshold proved to be necessary for these samples, [37]. The interpretation of each De distribution, is based on its overdispersion, internal patterns and geological observations. Consequently, either a mixture model (Finite Mixture Model), [45] or a central model (Central Age Model, [46] (sup mat) has been chosen.

The dose rate (Dr) is the sum of the contribution from cosmic, gamma and beta Dr. Alpha Dr is considered negligible since we assume that the outer layer of each grain irradiated by alpha particles has been HF etched. The cosmic Dr has been calculated from the equation of Prescott and Hutton [47]. It is mainly driven by the geographic coordinates of the sample and its burial depth. The gamma Dr had been measured in the field with a portable gamma spectrometer: the gamma probe was inserted 30 cm deep into the stratigraphic section, at the location where the sediment sample had been taken out. The gamma spectra were analyzed with the threshold technique [11, 35]. The beta Dr has been calculated from the radioisotopic contents (^238^ U series, ^232^ Th series and ^40^ K) of each sample, determined in the laboratory with high resolution gamma spectrometry (sup mat). Dose-rate conversion factors from Guérin et al, [48] and attenuation factor from Guérin et al, [49, 50], were applied. Correction for water content of the beta and gamma Dr was made using the coefficients of Zimmerman, [51], and assuming a past mean water content of 50+-15% of the saturated content (estimated 20 to 30% for the silty-sandy sediments of the Falémé). Details for the dose rate results are given in the supplementary material.

### 2.3. Sedimentary and stratigraphic contexts

**Fig 1:**
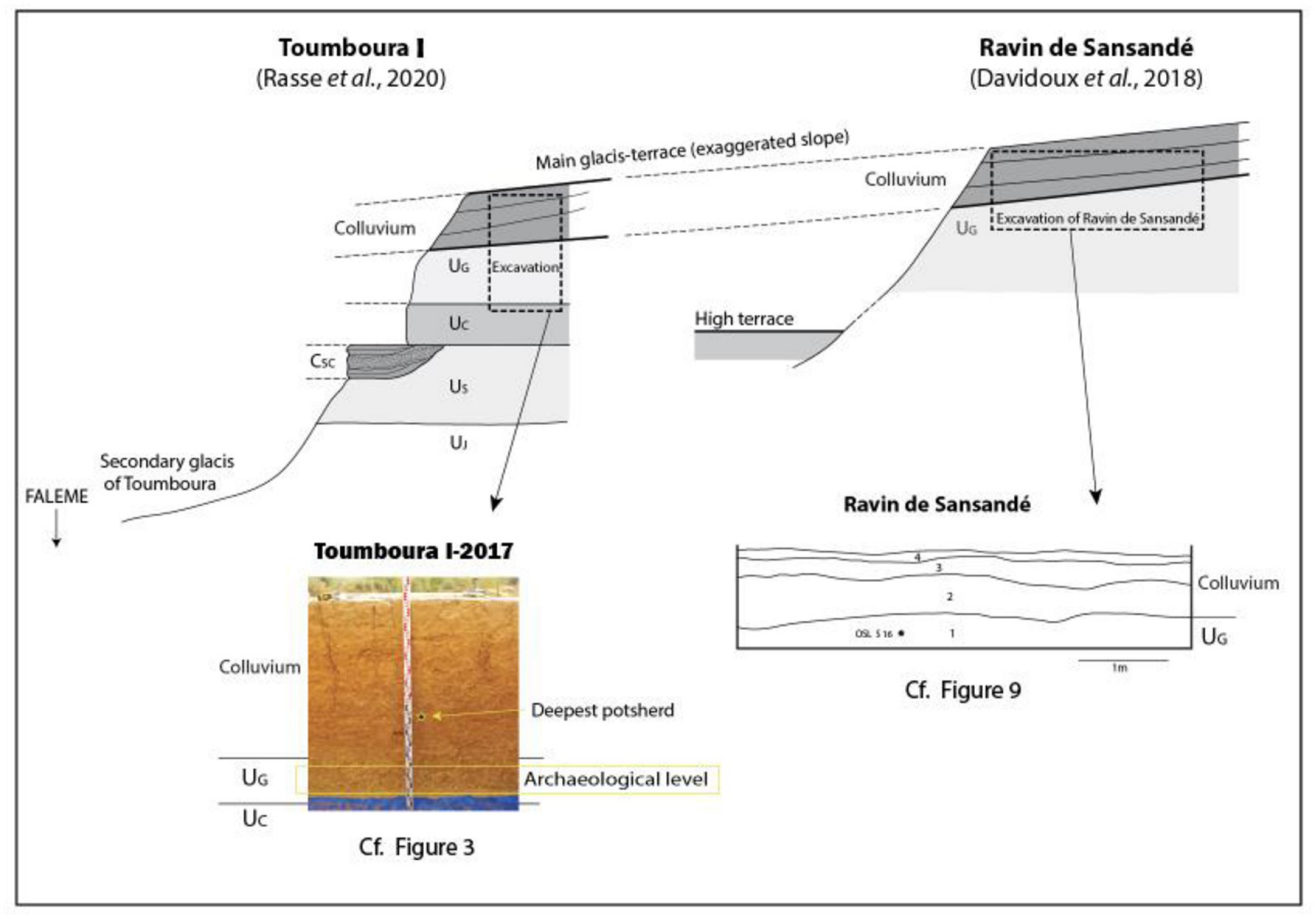
Summary of stratigraphic sections from the Toumboura, TMBI-2017 and Ravin de Sansandé sites.

Identification of stratigraphic units was made through sedimentary descriptions of sections and geomorphological field analyses. The sedimentary and stratigraphic contexts in the Falémé Valley show a combination of eolian inputs, alluvial processes through inputs from the Falémé river and its tributaries, and colluvial processes through the lateral transfer of soil particles and eolian dust on gentle slopes [40]. The Upper Pleistocene stratigraphic units visible at the TMBI-2017 and RDS sites contain archaeological levels composed primarily of Later Stone Age lithic industries (Fig 1). General summaries of the sedimentary contexts have been provided in Rasse and colleagues [40].

## 3. The Later Stoner Age at Toumboura I-2017 site

The Toumboura archaeological complex is located on the left bank of the Falémé river, approximately 500 meters south of the present-day village of Toumboura (13°57’17.6" N, 12°12’47.0" W). Archaeological and geomorphological research conducted in 2014 established a reference stratigraphic section for the area. This section, named Toumboura I, is 5.50 m high and serves as a reference for the description of the sedimentary units of the TMBI-2017 site, excavated in 2017, a few tens of centimeters back from the Toumboura I section. LSA lithic materials were identified during the cleaning of the Toumboura I section cut in 2014, and showed the presence of an industry essentially composed of small flakes, blades, and bladelets, as well as microlithic tools in the form of geometric pieces and segments [22, 23]. The coherence of the sedimentary, chronological and cultural data observed in the Toumboura I section motivated the extension of the excavation (Toumboura I-2017) in order to collect a more extensive lithic corpus.

### 3.1. Sedimentary units and chronology of the Toumboura reference section

**Fig 2:**
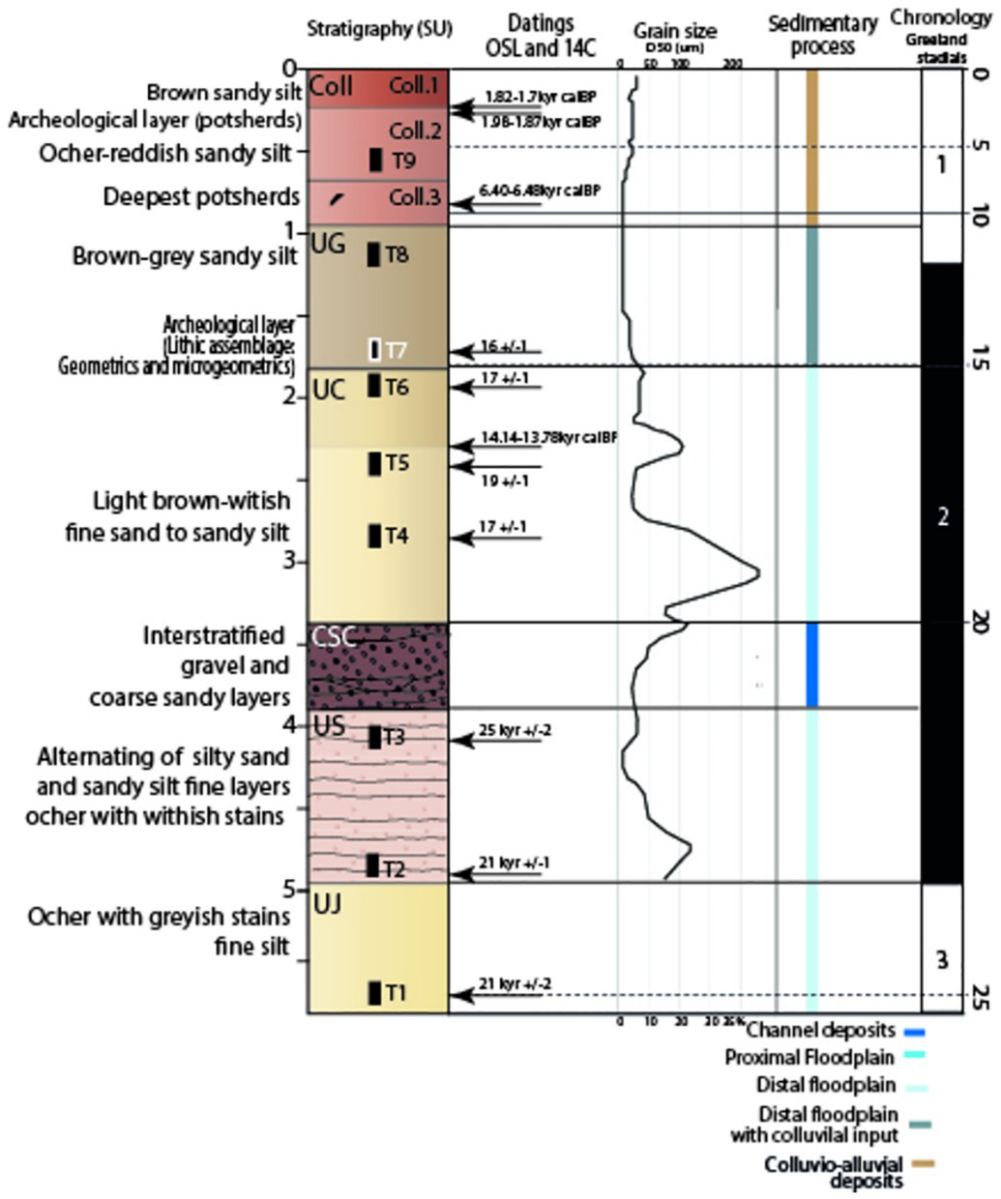
Sedimentary, stratigraphic, and chronocultural synthesis of Toumboura I and Toumboura- 2017.

The Toumboura I section presents 6 stratigraphic units that are used as a reference for the description of the entire sedimentary formations of the valley floor and which allowed the chronostratigraphic framework for the Upper Pleistocene and the transition to the Holocene to be set for the region (Fig 2) [40]. The section as a whole shows two silty packages, which compose units U_J_ and U_S_ at the base and U_G_ and U_C_ at the top, separated by a coarse channel level (C_SC_).

Unit U_J_ constitutes the base of the sequence. At Toumboura I, an OSL age suggests a date (sample T1) to around 21 ± 2 ka, but all the ages available at Toumboura [52] and particularly those obtained on Us at Toumboura I suggest that this sedimentation ends in the lower part of the date obtained around 23 ka. This unit is homogeneous, silty-clayey, yellow-brown in color with whitish patches that reflects low-energy fluvial transport presumably fed by eolian inputs. The presence of ochre and grey patches indicates redox pedogenesis processes, as during temporary waterlogging. Lower down in the Toumboura sequence, an MSA assemblage has been described in this unit where U_J_ has been dated between 40 ± 3 ka and 30 ± 3 ka [52].

The overlying U_S_ unit formed after an erosional phase as shown by the sharp boundary truncating the U_J_ deposits. U_S_ is dated by two OSL samples (sample T2 and T3), at 21 ± 1 ka, and 25 ± 2 ka, respectively. U_J_ is dated between 27 and 20 ka at the beginning of isotopic stage 2 (MIS 2) and corresponds to a bedded unit showing regular alternation of fine sands, white silts and coarser ochre sands. The often-erosive contact between the different beds as well as the coarser grain size indicate deposition by moderately energetic alluvial flows. The soil features are not very pronounced and suggest weak vegetation on a broad alluvial plain subject to flooding and wind action. The overlying C_SC_ unit is in erosive contact with U_S_. The sediments are composed of heterometric (millimetric to centimetric) rolled pebbles, set in a matrix of highly cemented fine red sands. This deposit testifies to one or more energetic torrential-type episodes.

U_C_ overlies the channel deposits of C_SC_. This unit is well dated by three OSL ages (samples T4, T5, T6), between 17 ± 1 ka and 19 ± 1 ka respectively. A radiocarbon age obtained on a charcoal found between samples T5 and T6 is chronostratigraphically reversed (14.135–13.780 cal BC). U_C_ is a very compact unit, generally composed of whitish beige sandy silts. It has a sandier base (fine to medium sands) and a small sandy pass at the level of the sampled charcoal. It is structured in small angular to subangular aggregates and contains oxidation stains and ferro-manganic concretions. U_C_ testifies an alluvial overflow deposit on a broad floodplain marked by more energetic fluvial deposition, particularly at the base, and then by the development of pedogenesis.

The abundance of rusty and ochre nodules suggests an increase in lateral erosive inputs, mainly by runoff at the expense of the glacis-terraces.

The last unit corresponds to the colluvium that formed the cover of the glacis where phases of erosion and accumulation alternated. It is composed of three sub-units. The first colluvial phase consists of beige silts reminiscent of U_G_ but the slightly gullying lower limit shows a reworking of the earlier formations. It is dated by radiocarbon to 6396–6298 cal BC. The second contains reddish-ochre silts with small black concretions.

It testifies to the erosion of well-developed ferralitic soils but is also characterized by a structure of powdery lumpy aggregates indicating active pedogenesis. Neolithic ceramics were found in this level. Finally, the upper sub-unit of the section is composed of compact brown-beige silts that constitute the last pedogenized colluvial unit undergoing erosion. We observed that 34 cm of sediments were eroded between 2014 and 2018 from this surface sub-unit. It is dated by two charcoals to the first millennium AD. This colluvial unit, as a whole, attests to the increase in colluvial dynamics and pedogenetic processes at the expense of alluvial dynamics.

Table 1 shows several inversions in the ages obtained by OSL and radiocarbon analyses, but globally, the dating of the reference section is robust (Table 1). It mainly covers MIS 2, with units U_S_ to U_G_ dated between 21 ± 1 ka and 16 ± 1 ka. The ages of T6 (unit U_C_) and T7 (unit U_G_)—the latter corresponding to the LSA archaeological level- are slightly older (17 ± 1 and 16 ± 1 ka) than the ^14^C dated charcoal (12163–11854 cal BC) also collected in unit U_C_. It is possible that bioturbations have affected either samples T6 and T7 (see sup mat) or the positioning of the charcoal. However, the beginning of the transition to the African Humid Optimum has not been documented in both cases. The humid time span in the African Sahel, known as the African Humid Optimum period, occurred in this region after the Last glacial period and lasted from ca. 14.5 to 5 ka, with an optimum between 11 and 6 ka [53].The sedimentary analyses described above also support this attribution.

**Table 1:** OSL and radiocarbon ages obtained for the sedimentary units at Toumboura I. Toumboura I-2017 was excavated from the top of the colluvium down to U_G_ sample T7 represents age provided directly from the Later Stone Age archaeological horizon. SU: Sedimentary Unit.

SU	Depth (cm)	Samples	Materials	14C ±1δ BP age	Calibrated age 2δ Cal BC/AD	Dose equivalent (Gy)	Dose rate (Gy/ka)	Age (ka)	Cultural chronology
Coll	- 25 cm	ETH-87705	Charcoal	1833 ± 23	125–243 calAD				
Coll	- 28 cm	ETH-87707	Charcoal	1281 ± 23	671–770 calAD				
Coll	- 30 cm	ETH-87706	Charcoal	1966 ± 23	38 BC—80 calAD				
Coll	- 70 cm	T9	Sediment				1.91 ± 0.10		
Coll	- 83 cm	ETH-87708	Charcoal	5556 ± 26	6396–6298 calBC				
U_G_	- 100 cm	T8	Sediment				2.18 ± 0.11		
U_G_	- 170 cm	T7	Sediment			38.0 ± 1.4	2.32 ± 0.12	16 ± 1	LSA
U_c_	- 190 cm	T6	Sediment			38.9 ± 1.4	2.29 ± 0.12	17 ± 1	LSA
U_C_	- 230 cm	ETH-55080	Charcoal	12114 ± 59	12163–11854 calBC				
U_C_	- 310 cm	T5	Sediment			39.7 ± 1.7	2.10 ± 0.11	19 ± 1	
U_C_	- 350 cm	T4	Sediment			35.5 ± 1.6	2.11 ± 0.12	17 ± 1	
U_S_	- 380 cm	T3	Sediment			37.3 ± 1.6	1.52 ± 0.07	25 ± 2	
U_S_	- 480 cm	T2	Sediment			28.3 ± 0.9	1.32 ± 0.06	21 ± 1	
U_J_	- 630 cm	T1	Sediment			44.8 ± 1.1	2.11± 0.11	21 ± 1	

The base of unit U_G_ is dated by an OSL age (samples T7) at 16 ± 1 ka. Sample T8 is, according to the single-grain analysis, considered too bioturbated to be dated. The sedimentation shows a progressive change from Uc to U_G_ and the base of U_G_ consists of a first transitional horizon, and includes the LSA archaeological assemblage at Toumboura I and TMBI-2017, associated with sample OSL T7. U_C_ testifies an alluvial overflow deposit on a broad floodplain marked by more energetic fluvial deposition, particularly at the base, and then by the development of a fine paleosol in an alluvial plain.

### 3.2. The excavation of Toumboura I-2017

**Fig 3:**
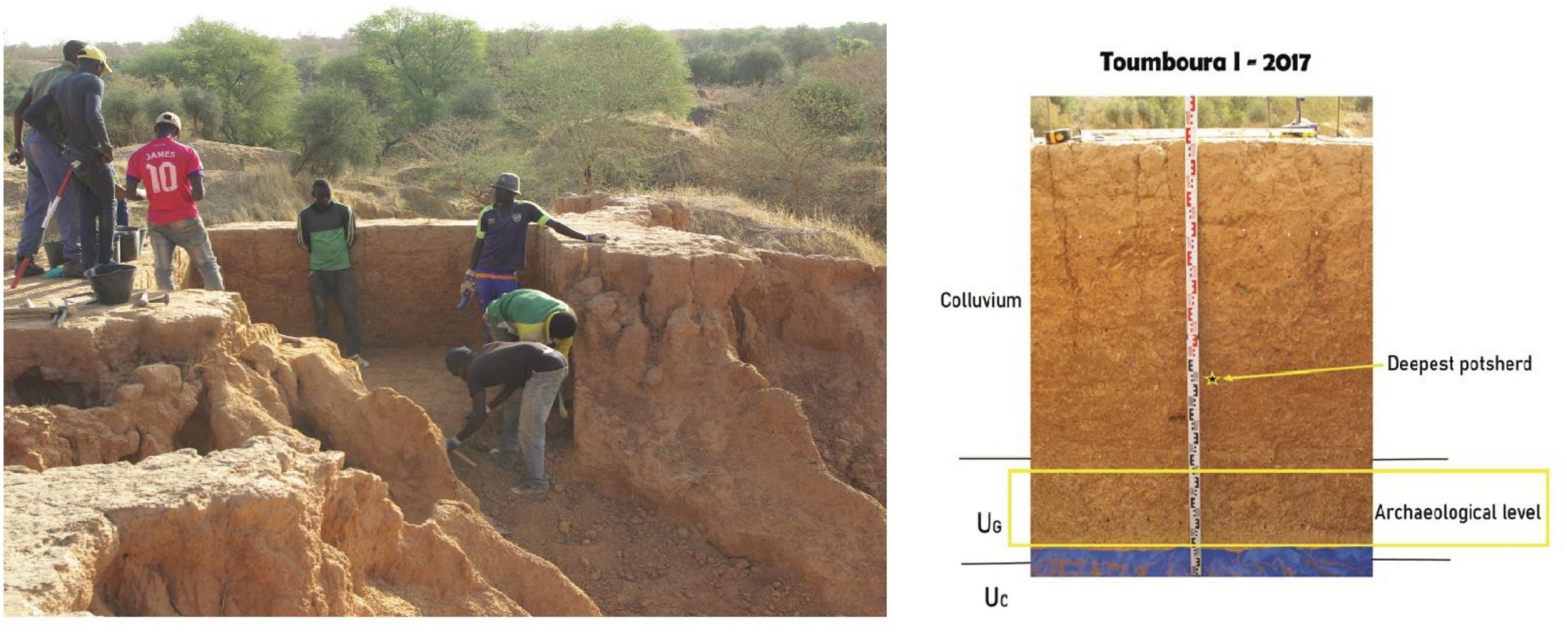
The site of TMBI-2017. (A) View of the excavation from the South. (B) Stratigraphic sequence from E-W section.

In 2017, the excavation was extended on the top part of the glacis, a few centimeters back from the 2014 Toumboura I reference section (Fig 3). This extension was named Toumboura I-2017 (13°57’17.62" N, 12°12’47.12" W). An area of 6 m^2^ was excavated to a sterile soil at a depth of -1.70 m below from the surface (Table 2). Arbitrary spits of 20 cm were chosen for the upper colluvium while the excavation of the lower sedimentary unit U_G_ (30 cm of thickness) allowed us to document an important lithic industry in good state of preservation. Artifacts were found at all levels. A total of n = 6583 artifacts was collected. The upper colluvium remobilized a heterogeneous assemblage of ceramic sherds (n = 13), rolled lithic artifacts (n = 4364), and a small hematite axe. The lowest colluvium sub-unit, materialized by a layer of silty sediments to fine brown-grey sands with concretions, yielded a rather scattered but well-preserved lithic industry (n = 504). The main archaeological level is found in the underlying unit U_G_ that provided a lithic assemblage (n = 1701) of LSA type described here.

**Table 2:** Summary of artefacts finds per sedimentary unit at Toumboura I-2017.

Sedimentary units at Toumboura I-2017	Depth in cm with units	Deposit types	Correspondence with TMBI sedimentary units	Lithics artifacts	Ceramics artifacts	Cultural chronology
Coll. 1 & 2	0–83 cm	Recent colluvium	COLL.	4364	13	Recent times
Coll. 3	83–140 cm	Distal floodplain deposit with colluvial input		504		Neolithic ceramic
UG	140–170 cm	Compact silts	UG	1701		Late Stone Age

### 3.3. Lithic industry of Toumboura I-2017

#### 3.3.1. Assemblage composition and sampling

The LSA lithic industry is composed of n = 1701 artifacts including n = 17 cores, n = 793 end-products and tools, and n = 891 knapping waste artifacts (Table 3).

**Table 3:** Composition of the Late Stone Age lithic assemblage at Toumboura I-2017.

	Chert	Greywacke	Quartz	N =	Total %
Cores	9	3	5	17	0.99
Flakes	168	58	81	307	18.04
Semi-cortical flakes	29	12	7	48	2.82
*Débordant* flakes	59	18	15	92	5.4
Incomplete flakes	38	5	8	51	2.99
Blades	22	12	2	36	2.11
Semi-cortical blades	17	3	4	24	1.41
Flank blades	13	4	2	19	1.11
Bladelets	143	23	9	175	10.28
Segments	34		5	39	2.29
Backed bladelets	2			2	0.11
Blocks with removals scars	15		3	18	1.05
Distal fragments	29	1	5	35	2.05
Proximal fragments	33	8	6	47	2.76
Small flakes ≤ 20 mm	71	8	14	93	5.46
Flakes ≤ 10 mm	132	35	54	221	12.99
Angular waste	304	85	88	477	28.04
Total	1118	275	308	1701	100%

#### 3.3.2. Raw materials

A wide variety of raw materials are represented in the lithic assemblage, differing in technical properties and knapping suitability. Chert dominates (65.72% of the total assemblage), followed by quartz (18.12%) and greywacke (16.16%). Several chert outcrops are known on both sides of the Falémé river. This highly siliceous material is present in the greywackes that form the bedrock in the study area. Greywacke and chert are present in (sub-) primary outcrops in the form of veins and boulders in many localities around the site. Quartz is available in abundance as small to medium-sized alluvial pebbles on the banks of the Falémé River, while quartz veins are more prevalent on the right bank of the Falémé, opposite to TMBI-2017. These materials are therefore available within a relatively close radius to the site, although the chert appears more sporadically in the landscape and involves a more constrained acquisition process. Because of its good fracturing properties, the exploitation of chert provided the most diagnostic techno-typological elements in regards to the extent of the technical knowledge of the knappers of TMBI-2017 and shows the importance of the production of flakes and bladelets. Conversely, quartz provided proportionally more flakes than bladelets, possibly due to the intrinsic constraints of this material. In general, whatever the material selected, the concomitant production of flakes, blades and bladelets products is observed. Greywacke, on the other hand, was used infrequently for such a high quality material found in relative abundance in the direct vicinity of the site.

#### 3.3.3. Cores

The reduction processes of the 17 cores in the assemblage shows correlation between cores sizes and raw materials on the one hand, and between reduction modality / blank production / number of striking platforms on the other hand (Table 4). Chert cores (n = 9) were greatly exploited (i.e until exhaustion) than quartz (n = 5) and greywacke (n = 3) cores from which only few blanks were removed before abandonment. Indeed, the number of blanks removals observed on chert cores (n = 38) is greater than those identified on quartz (n = 16) and greywacke cores (n = 9). Six cores show the removal of one blank, on four chert cores, one quartz and one greywacke core. However, five cores showed removals with two blanks out of three chert and two quartz cores. The discontinuous removal of blanks per raw material presents a real problem for knappers who make and use stone tools, especially when they are mobile. Technical discontinuities are obvious when raw materials don’t allow enough striking platforms to remove more than one blank. The strategy of producing two blanks per raw materials when a need presented itself maybe would result of the nodules abundance. This situation creates masses of nodules and blanks, which make sites easier to identify on the landscape. It is not unreasonable to expect that the blanks removals of raw materials also play a role in the final size and shape of artifacts produced. The hypothesis relating to the removal of one or two blanks from the raw materials is a dynamic process linked to the choice of knappers in relation to the types of blanks or tools to be produced and according to the availability and quality of the nodules which can help to produce it [29, 31, 54, 55].

**Table 4:** Cores attributes, Toumboura I-2017 site. (A) Dimensions of cores per raw material. (B) Cores reductions modalities per core main production objectives and numbers of striking platforms.

**A.**		Length (mm)				Width (mm)				Thickness (mm)			
	N =	Min.	Max.	Mean	St. dev.	Min.	Max.	Mean	St. dev.	Min.	Max.	Mean	St. dev.
Chert	9	33	37	34	1.01	25	31	27	1.41	25	30	27	1.18
Quartz	5	29	32	30	0.95	21	27	23	1.9	18	21	19	0.96
Greywacke	3	34	39	36	1.91	26	34	29	3.31	28	33	28	2.53
**B.**	Cores with bladelet removals (n =)			Cores with flake removals (n =)		Cores with blade removals (n =)		Cores with mixed blank removals (n =)		One striking platform (n =)		Two striking platforms (n =)	
Frontal	6			3		2		1		12			
Semi-rotating	2							1		2		1	
Peripheral	1			1						1		1	
Total	9			4		2		2		15		2	

**Fig 4:**
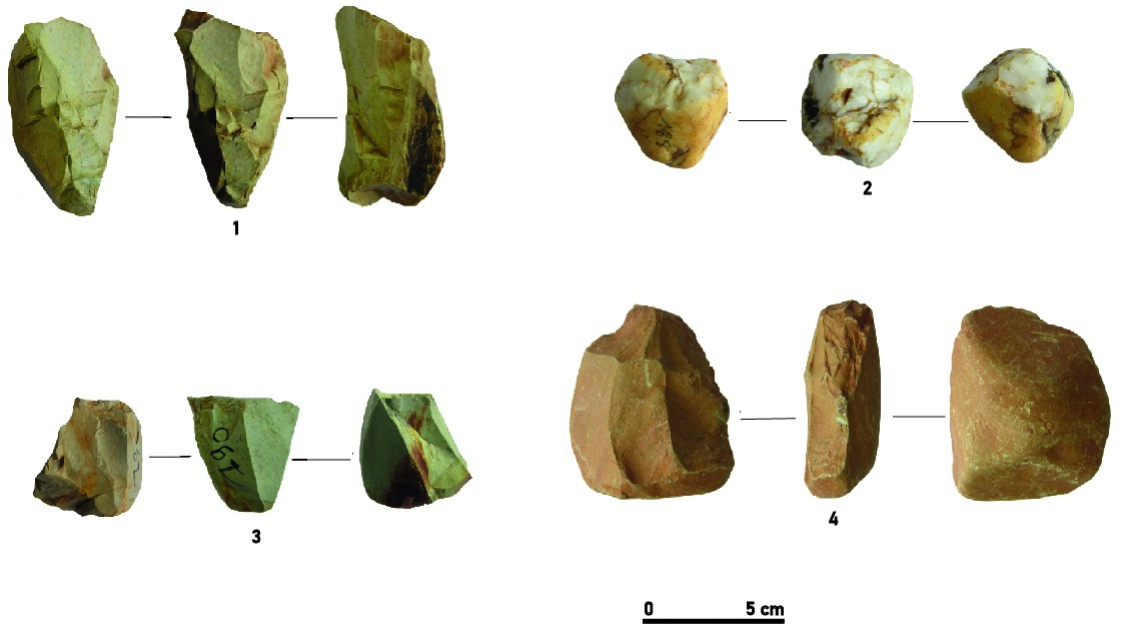
Different core exploitation modalities at Toumboura I-2017. (1) peripheral core, chert. (2) frontal core, quartz. (3) semi-rotating core, chert. (4) frontal core, greywacke.

The cores of the assemblage show varied exploitation modalities, dominated by frontal exploitation of the volumes (n = 12), and more rarely by semi-rotating (n = 3) or peripheral (n = 2) core reduction (Table 4). Their exploitation was aimed at the production of flakes as well as bladelets and more rarely of blades. While five frontal cores were clearly used for flake (n = 3) and blade (n = 2) productions, the detachment of bladelets was observed on cores with frontal (n = 6), semi-rotating (n = 2) and peripheral (n = 1) core reduction patterns. However, two cores (frontal and semi-rotating) showed mixed removal of flakes and bladelets. (Fig 4).

Generally, chert cores present unidirectional (n = 7) and, rarely bidirectional (n = 2) exploitations with and their striking platform being often plain. Chert cores (n = 4) testify to the detachment of blanks bearing cortical surfaces, suggesting the choice of small pebbles. The production on the chert cores appears slightly more standardized and characterized by a higher frequency of flakes and bladelets removal, compared to the other knapped materials. The exploitation of quartz shows a significant variability and is mainly oriented towards the production of a small number of semi-cortical flakes. It is collected in the form of small pebbles rolled by the Falémé river, often having undergone several shocks and substantial weathering. The reduction scheme for quartz cores is based on the recurrent reorientation of the cores by exploiting the cortical surfaces as striking platforms. Greywacke cores are larger in size, and they provided, at the beginning of the reduction sequence, relatively thicker flakes and blades. There are abundant greywacke outcrops in the mid-valley, mainly in the form of tablets up to several dozen centimeters in length. The intensity of rainfall during the winter season contributes to the erosion of the primary sources of greywacke, making blocks of this raw material accessible at every point in the valley.

#### 3.3.4. Flakes, blades and bladelets

**Fig 5:**
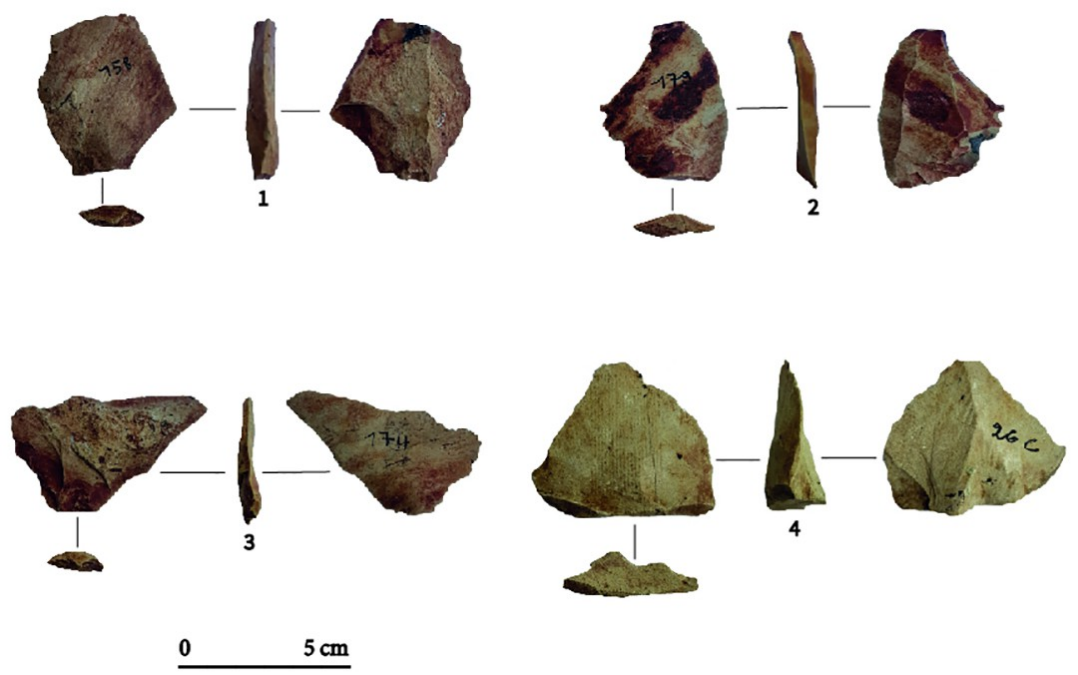
Flake blanks at Toumboura I-2017 site. (1) *Débordant* flake, greywacke. (2) Simple flake, chert. (3) Semi-cortical flake, chert. (4) Simple flake, greywacke.

There are n = 498 flakes in the studied sample, made on chert (59%), quartz (22.2%) and greywacke (18.6%), with small dimensions on average (Fig 5; Table 6). Although fully cortical flakes related to the initial stage of reduction of raw material are not numerous, n = 48. Semi-cortical flakes have generally facetted (n = 34) or plain (n = 14) platforms. Most of the flakes are simple flakes (n = 307) but *débordant* flakes (n = 92) are also numerous and testify to the stages of preparation or maintenance of the flanks of the core. Conversely, the cortex-free flakes seem to attest to a main production phase, generally from a frontal exploitation of the cores with a well-developed striking platform. Some flake fragments appear to have broken during core reduction (n = 51) at the proximal (n = 45) or distal part (n = 23). Siret fractures (n = 6) are also present, probably indicating both the use of hard hammer percussion and the quality of raw material. Flakes are produced during the preparation and maintenance stages of reduction but also as intended end-products.

**Fig 6:**
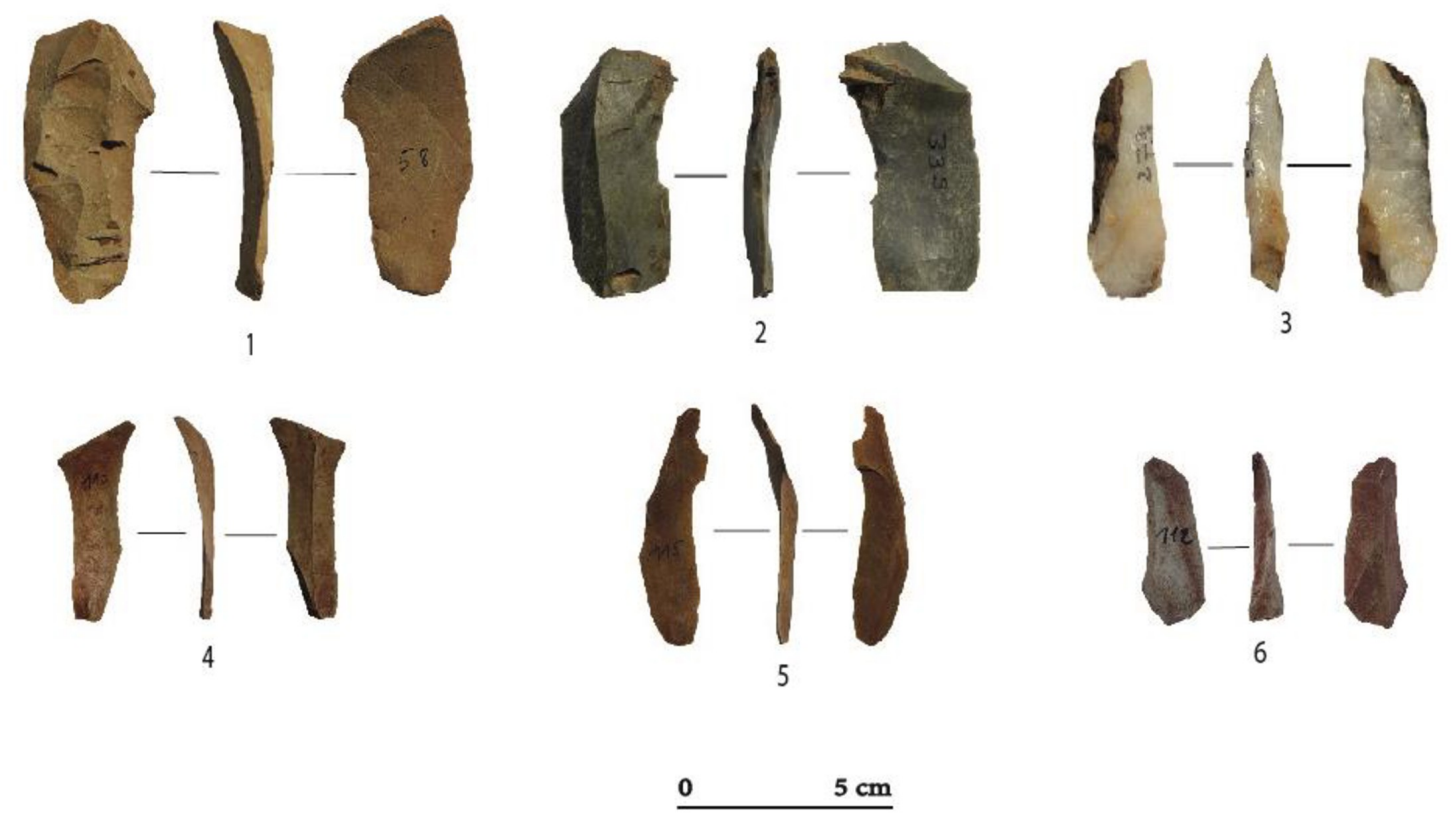
Blade and bladelet blanks at Toumboura I-2017 site. (1) Blade, chert. (2) Semi-cortical blade, chert. (3) Flank blade, quartz. (4, 5, 6) Bladelet, chert.

Blades (n = 79) often have irregular morphologies and converging or parallel edges (Fig 6). They have straight (n = 42), concave (n = 23) and convex (n = 14) profiles, with mostly plain (n = 54), but also linear (n = 17) or punctiform (n = 8) platforms. Blades are highly variable in size, with mean dimensions of 38 mm long, 24 mm wide and 8 mm thick (Table 5). Non-cortical blades (n = 36) are the most common form. However, n = 24 blades show cortical surfaces on the distal (n = 7), lateral (n = 11), bilateral (n = 4) and mesial (n = 2) portions. A total of n = 19 flank blades were obtained, in most cases, from frontal cores, by exploiting the lateral parts of the exploitation surfaces.

**Table 5:** Segments. Types and numbers of blanks used per raw materials.

Segments	Chert	Quartz	N =
Flakes	9	2	11
Bladelets	21	3	24
undetermined	4		4
Total	34	5	39

Bladelets are much more numerous (n = 175) and are all ≤ 12 mm wide (Table 6). They are mainly obtained from chert (81.7%) and to a lesser extent from greywacke (13.14%) and quartz (5.14%). The bladelets have concave or convex profiles with, in most cases, triangular or irregular sections (Fig 6). They are produced by unidirectional or oblique knapping on cores with frontal, semi-rotating and rarely peripheral core reduction modalities. Their platforms are plain (n = 145) and rarely linear (n = 23) or punctiform (n = 7). Blades and bladelets are produced on small cores with minimally prepared striking platforms.

**Table 6:** Dimensions of flakes, blades, bladelets, segments and backed bladelets per raw material at Toumboura I-2017.

			Length (mm)				Width (mm)				Thickness (mm)			
		N =	Min	Max.	Mean.	St.dev	Min.	Max.	Mean.	St.dev	Min.	max.	Mean.	St.dev.
Flakes N = 498	Chert	294	20	48	29	1.21	18	39	21	1.06	2	20	9	0.74
	Quartz	111	32	64	29	3.33	14	36	23	1.49	3	18	11	1.01
	Greywacke	93	24	69	30	4.73	15	41	26	3.06	3	29	13	2.93
Blades N = 79	Chert	52	29	54	35	2.76	13	24	15	1.27	2	12	7	0.98
	Quartz	8	26	52	42	6.67	13	26	25	4.45	3	10	9	6.87
	Greywacke	19	28	53	39	6	14	23	21	2.84	2	19	9	4.12
Bladelets N = 175	Chert	143	21	49	32	1.69	7	12	10	0.31	2	12	8,32	0.6
	Quartz	9	21	37	29	3.77	7	12	9	1.19	3	12	7,22	2.12
	Greywacke	23	27	46	28	4.3	6	12	10	1.01	2	11	7,42	2.19
Segments N = 39	Chert	34	12	17	14	1.57	5	10	7	1.77	3	6	4	0.97
	Quartz	5	13	16	14	1.25	7	9	8	1.14	3	5	4	0.25
Backed bladelets N = 2	Chert	2	22	23	22	0.5	9	10	9	0.12	4	5	4	0.5

#### 3.3.5. Tools

**Fig 7:**
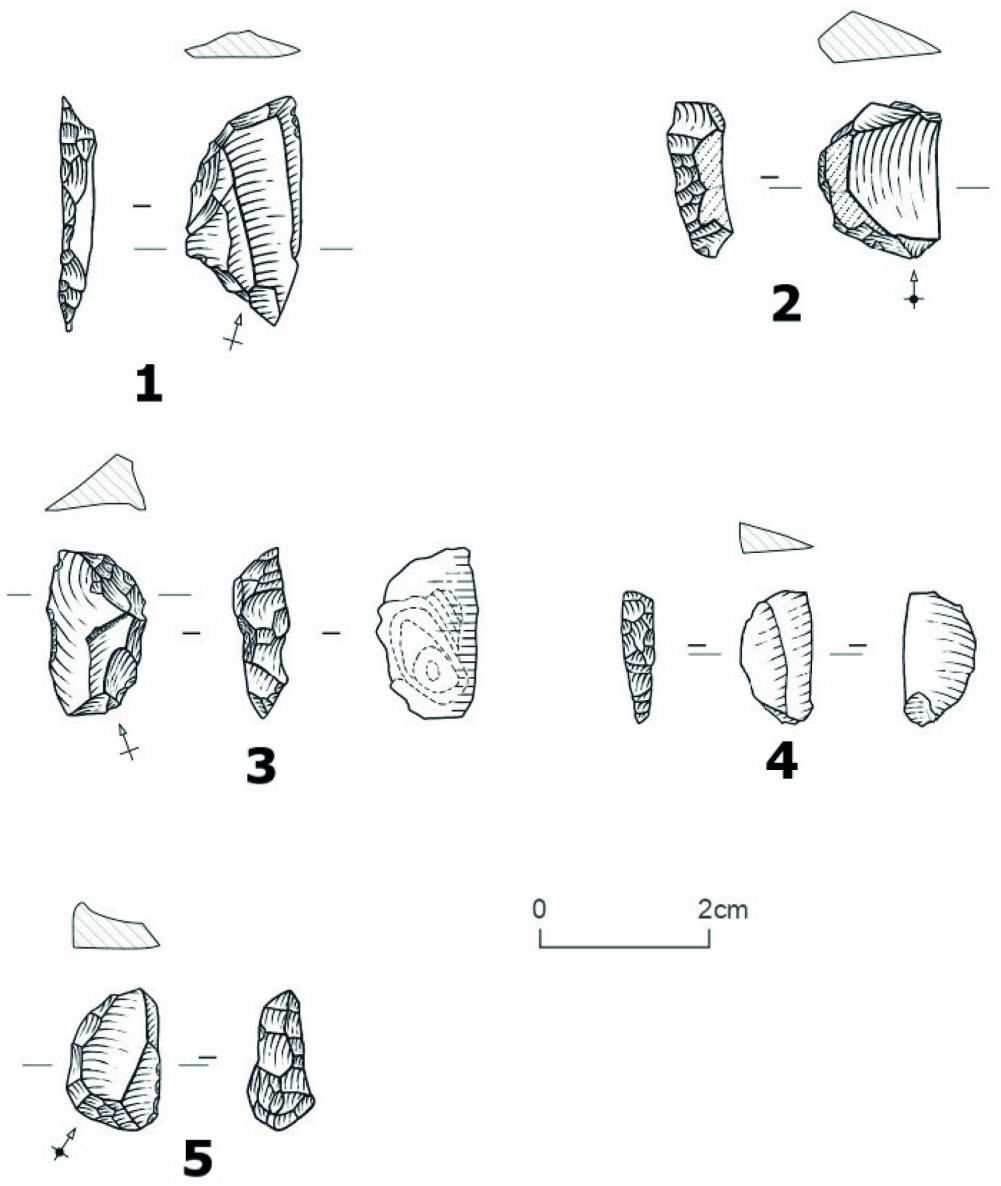
Example of segments at Toumboura I-2017. (1) On bladelet. (2, 3, 5) On flakes. (4) On undetermined blank. (1, 3, 5) Segments with discontinuous retouch and irregular cutting edges. (2, 4) Segments with abrupt retouch and straight cutting edge. (1, 2, 3, 4) Chert. (5) Quartz.

At TMBI-2017, the toolkit is minimally not very diversified. It is composed of 39 segments and two backed bladelets (Table 5). However, they are varied in terms of blanks, raw material, type, and location of retouch. Segments are made either on flakes (n = 9), on bladelets (n = 21), or on undetermined blanks (n = 4) (Fig 7) and are both on chert (n = 34) and quartz (n = 5) (Table 5). Generally, the segments have a convex edge with an abrupt continuous retouch, opposite to a straight unretouched edge. However a total of 13 segments show discontinuous retouch and slightly broken or irregular cutting edges. Two quartz segments stand out with semi-abrupt retouch. The two backed bladelets are made of chert and are characterized by marginal abrupt lateral retouch, with straight edges, and no modification of the distal or proximal ends. Tools are very few compared to flakes and bladelets production. The main goal of the debitage was to produce flakes and bladelets that only sporadically were retouched and turned into tools.

## 4. The Later Stone Age of the Ravin de Sansandé site

The RDS site (13°55’06.7’’ N, 12°12’53.1’’ W) is located less than 5 km south of the village of Sansandé. It lies more than 350 m from the Falémé river and more than 200 m south of the Sansandé seasonal gully, and dominates an ancient alluvial terrace attributed to the 1^er^ millennium AD [56]. Discovered in 2018 during paleo-environmental surveys, the site had three particularly dense lithic concentration piles on the surface, associated with a few ceramic sherds. One test-excavation and two excavation campaigns were conducted between 2018 and 2020 on the site, on the highest point of the promontory, in order to investigate the stratigraphic deposit. Below the colluvial units containing reworked material of different periods and ceramic sherds, a lower unit containing a very well-preserved lithic industry, characteristic of the LSA, was discovered.

### 4.1. Excavations at the Ravin de Sansandé

In 2018, a north-trending test excavation (3 x 1 m) exposed sedimentary units from the surface through the transition between two archaeological strata labeled US2 to US1. The upper level US2 is composed of a mixed material of artifacts and ceramics. A 15 cm-thick transitional level was identified, containing ceramic material from US2 and a few LSA-type lithic artifacts, heralding the archaeological level of US1, which was not excavated to the end.

In 2019, another trench (5 x 2 m), oriented north-south, was opened 26 m from the 2018 test-excavation in order to evaluate the extension of the LSA archaeological horizon to the west. This excavation yielded two major sedimentary levels:

- US1 is composed of a thick colluvial layer about 60 cm deep, with heterogeneous archaeological material (lithics and ceramics) from protohistorical and recent periods;
- US 2 is a sandy-silty sediment horizon with the presence of a few reddish pisolite gravels and sparse LSA-type lithic material with abundant lithic debris.

Finally, in 2020, an east-west oriented trench (10 x 1.5 m), interrupted by a 3 m berm in the western part was opened in order to link the two previous excavation areas of 2018 and 2019. The eastern part of the trench is 6 m long, beginning at the northern end of the 2018 excavation; while the western part of the trench extends 4 m from the southern end of the 2019 excavation.

The three excavation areas, exposing a total area of 25 m^2^, have broadly the same stratigraphic characteristics, including US1 containing the LSA artifacts at a thickness of 20 cm on average [41]. The knapping piles, partially *in situ*, uncovered by erosion in the gully area in the lower part of the promontory downstream from the site, also relates to this archaeological horizon, suggesting that the occupational surface was probably much larger. For all three excavation campaigns, we adopted arbitrary spits of 20 cm and the sediments were screened with a 2 mm mesh.

### 4.2. Sedimentary units and dating of the Ravin de Sansandé

**Fig 8:**
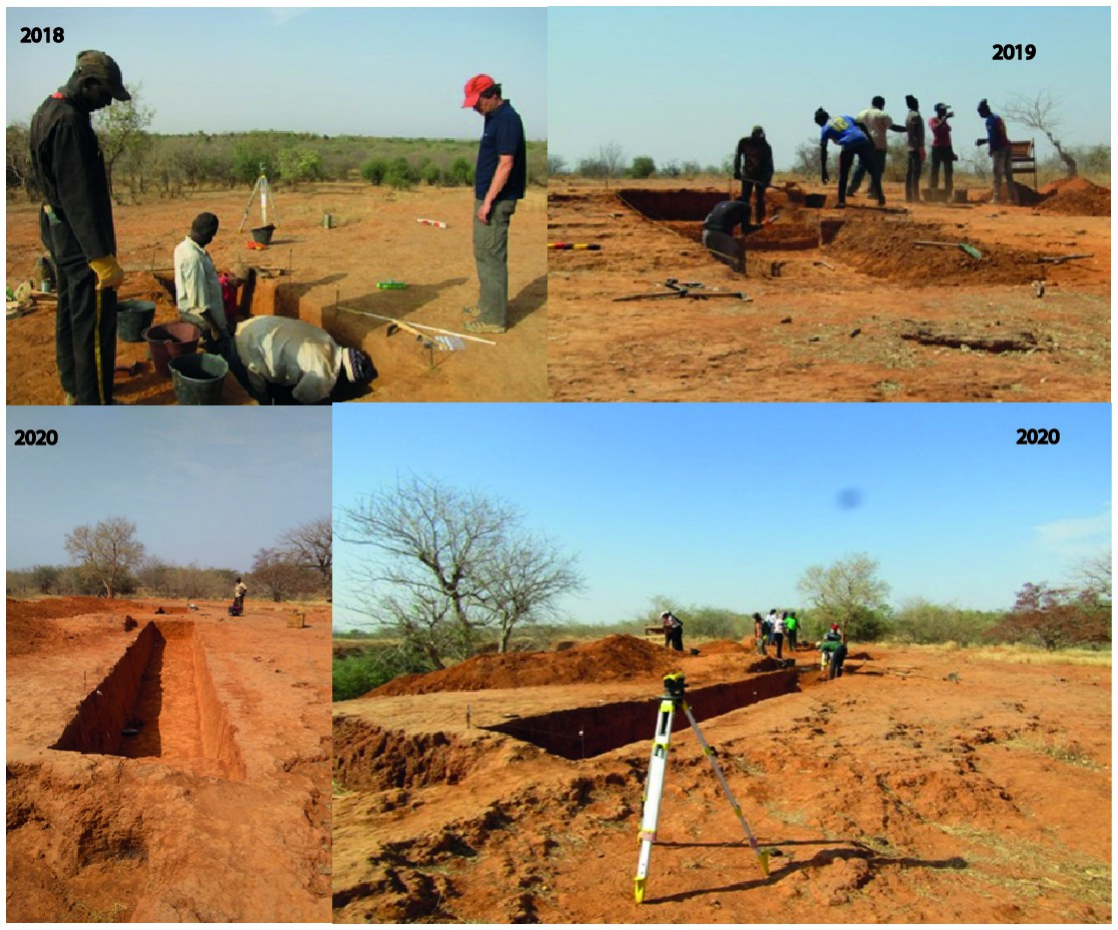
Global view of the three excavation campaigns at the Ravin de Sansandé site.

The sedimentary sequence observed in the RDS site section is characteristic of the Pleistocene to Holocene transition sediments in the Falémé Valley [22, 40]. The upper layer US4 is composed of a compact yellowish-grey silty sand. It yielded a heterogeneous lithic industry and rolled ceramic sherds. US4 corresponds to sub-actual sediments and testify to intense erosion of the glacis (Fig 8).

US3 also indicates a colluvial sequence but which underwent intense pedogenesis. It is composed of a homogeneous orange-red clayey silt with sub-angular half-centimetric aggregates. This colluvial layer is marked by the development of a ferralitic soil. It contains lithic artefacts, some of which are in a vertical position, as well as some ceramic sherds. The diversity of dates obtained on the charcoals (Table 6) likely indicates a cumulative soil that developed on regularly reactivated colluvium but under a dominant tree cover, between the 2^nd^ millennium BC and the last centuries, when the Falémé river was strongly incised [56].

Overlying US2 is a homogeneous but rather heterometric orange silty sand. It is a colluvial layer that mixes lithic and ceramic material. It corresponds to the establishment of a new colluvial formation, perhaps in response to the assertion of drier conditions in the second part of the Holocene, and to the incision of the Falémé River. It was dated to 5526–5380 calBC.

The lowermost sedimentary unit US1, in which the undisturbed Later Stone Age lithic material was found, corresponds to a sandy-silty deposit, rich in centimetric reddish pisolite gravels. These sediments were deposited by a lowly concentrated runoff on a glacis that was probably covered by open vegetation [56]. Unlike the Toumboura sequence, US1 developed in a context farther away from the banks of the Falémé river and is characterized by colluvial processes that developed on extensive glacis, bordering the alluvial plain.

Three ages are available for US1 (Table 7), from which two are obtained by OSL and differ from the one obtained by radiocarbon dating on charcoal. The fact that sedimentary unit US1 is characterized by a very weakly expressed pedogenesis allows us to consider dating from sedimentary samples by the OSL method as reliable, in contrast to the result on the charcoal that is more consistent with the ages of the upper units from which it could have percolated. The two sets of contents/activities are remarkably consistent in their homogeneity, especially since OSL samples S15 and S16 are 14 m apart and come from the same sedimentary unit. OSL ages of 13.0 ± 1.0 and 12.0 ± 1.1 ka indicate that the LSA at the Ravin de Sansandé site dates to the very end of the Pleistocene. Moreover, this age is consistent with those obtained for Pleistocene sedimentary accumulations observed about 200 m north of the site, dated at 20–10 ka [20]. US1 likely constitutes the upper part of these sedimentary accumulations.

**Table 7:** Results of radiocarbon and OSL analyses at the Ravin of Sansandé site per sedimentary unit (US). Depths indicated from the surface.

Sedimentary unit	Depth 2020	Samples	Materials	Age 14C ±1δ BP	Calibrated age 2δ Cal BC /AD	Dose equivalent (Gy)	Dose rate (Gy/ka)	Age (ka)
US4	0–20 cm							
US3	- 20–65 cm	ETH-87701	Charcoal	1126 ± 22 BP	1696–1918 calAD			
		ETH-87702	Charcoal	101 ± 22 BP	93–967 calAD			
		ETH-106235	Charcoal	1233 ± 22 BP	689–878 calAD			
		ETH-106234	Charcoal	3165 ± 23 BP	1498–1409 calBC			
US2	- 65–110 cm	ETH-106236	Charcoal	6504 ± 26 BP	5526–5380 calBC			
US1	-110–130 cm	ETH-87704	Charcoal	6217±27 BP	5300–5050 calBC			
		S15	Sediment			22.9 ± 0.7	1.76 ± 0.11	13.0 ± 1.0
		S16	Sediment			20.5 ± 0.8	1.71 ± 0.11	12.0 ± 1.1

### 4.3. The lithic industry of the Ravin de Sansandé

#### 4.3.1. Assemblage composition and sampling

The study presented here is based solely on the excavations carried out in 2020. These excavations led to the collection of 1708 artifacts, distributed over four stratigraphic units (Table 8). The material from the colluvium of US2 to US4 is not in primary position and was therefore excluded from the study. It consists of two bone fragments and ceramic sherds (n = 299), the latter being quite numerous at the top of the sequence, as well as lithic artifacts (n = 629), including backed pieces (n = 14).

**Table 8:** Composition of artifacts per stratigraphic unit at the Ravin de Sansandé site.

Stratigraphic units	Depth 2020	Lithics	Ceramics sherds	Bones fragments	N°
US 4	0–20 cm	362	196	2	560
US 3	- 20–65 cm	182	79		261
US 2	- 65–110 cm	85	24		109
US 1	-110–130 cm	778			778
**Total**		**1407**	**299**	**2**	**1708**

**Fig 9:**
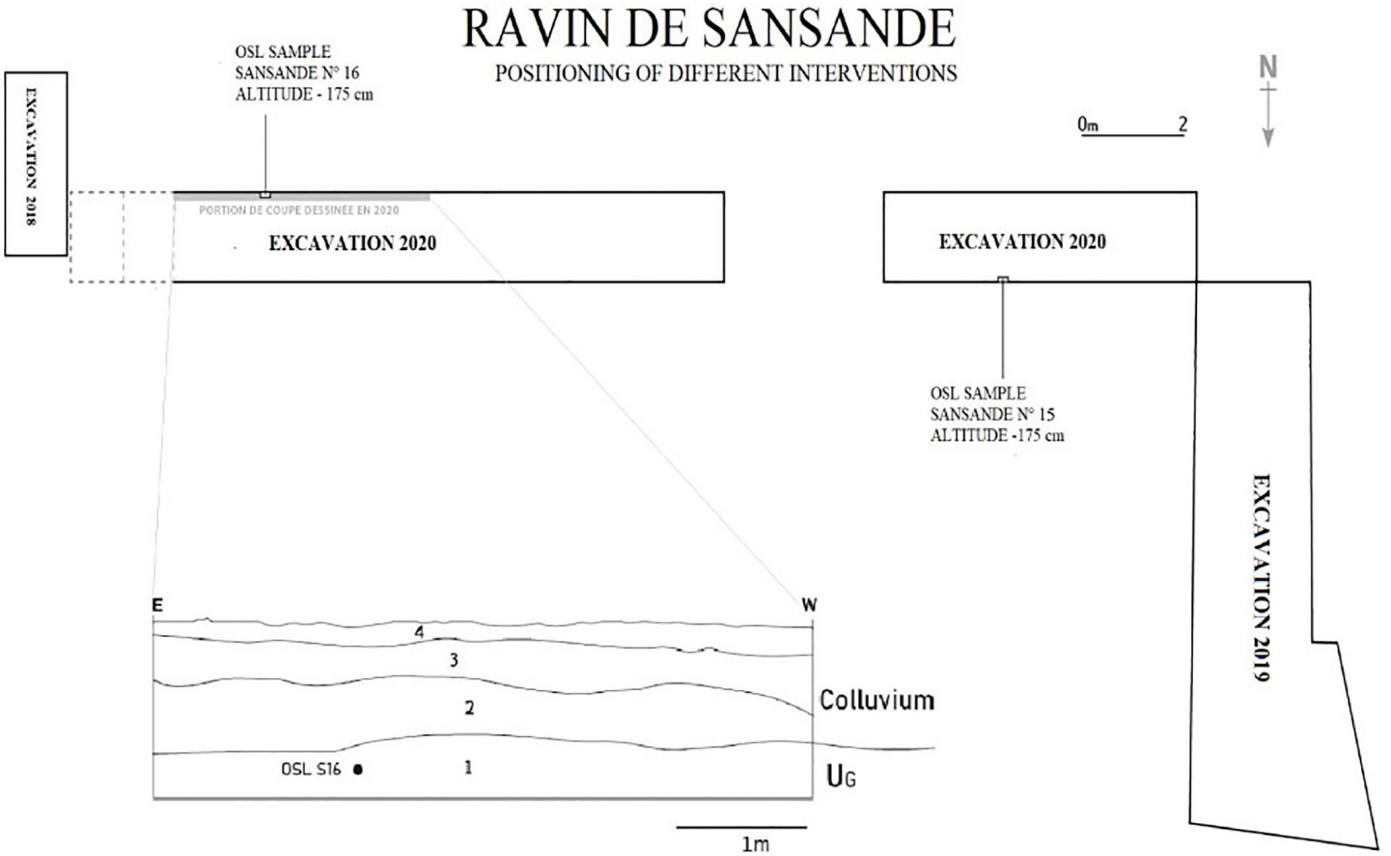
Schematics of the different interventions, stratigraphic units and OSL samples at the Ravin de Sansandé site.

Only the lithic material found in US1 in 2020 was selected for this study. The archaeological material was particularly concentrated in the areas where the sediment samples (S15 and S16) were taken for OSL dating (Fig 9).The LSA lithic assemblage of US1 is composed of n = 778 artifacts, including 574 diagnostic pieces and 204 undetermined pieces (Table 9). Only the 574 diagnostic pieces, including cores (n = 12), numerous flakes (n = 396), blades (n = 71), bladelets (n = 83), segments (n = 9) and a few end-scrapers (n = 3) were studied and analyzed in detail. The remainder of the assemblage (n = 204) is primarily composed of small blanks, such as fragmented pieces (flakes and bladelets) ≤10 mm, debris, and undeterminate pieces that who were counted and excluded from further description.

**Table 9:** Composition of the Late Stone Age lithic assemblage at Ravin of Sansandé, 2020 excavation.

	Chert	Greywacke	Quartz	Total N =	Total %
Cores	6	2	4	12	1.54
Flakes	187	49	7	243	31.23
Semi-cortical flakes	23	21	6	52	6.68
*Débordant* flakes	36	32	10	78	10.02
Laminar flakes	18	5		23	2.95
Blades	22	14	3	39	5.01
Semi-cortical blades	5	4		9	1.15
Flank blades	11	12		23	2.95
Bladelets	32	46	5	83	10.66
Segments	7	2		9	1.15
End-scrapers	2	1		3	0.38
Blocks with removals scars	9	7		16	2.05
Distal fragments	21	13		34	4.37
Proximal fragments	15	12	10	37	4.75
Small flakes ≤ 10 mm	26	19	3	48	6.16
Angular waste	31	33	7	69	8.86
Total	451	272	55	778	100%

#### 4.3.2. Raw materials

The lithic assemblage shows that the knappers used a variety of raw materials. Chert is well represented (61.32%), followed by greywacke (32.59%) and quartz (6.09%). As with TMBI-2017, all these materials are locally available and have different fracture qualities, from high (chert, greywacke) to moderate (quartz) quality. Although quartz has been knapped to obtain flakes, blades, bladelets, and tools, its lesser quality has produced many crushed and fractured blanks. The greywacke is often very fine-grained, presenting a good suitability for knapping. The dimensions of the pieces made of greywacke are larger than those of other materials, suggesting the availability of larger initial volumes for nodules. Some of the greywacke artifacts appear to be more prone to post-depositional chemical and mechanical weathering. Surface erosion of the pieces has led to the exposure of the laminated structure of this material. The chert appears more sporadically in the landscape and involves a more rigorous acquisition process. Because of its good aptitude for knapping, chert has provided the most diagnostic techno-typological elements concerning the extent of technical behavior.

#### 4.3.3. Cores

**Fig 10:**
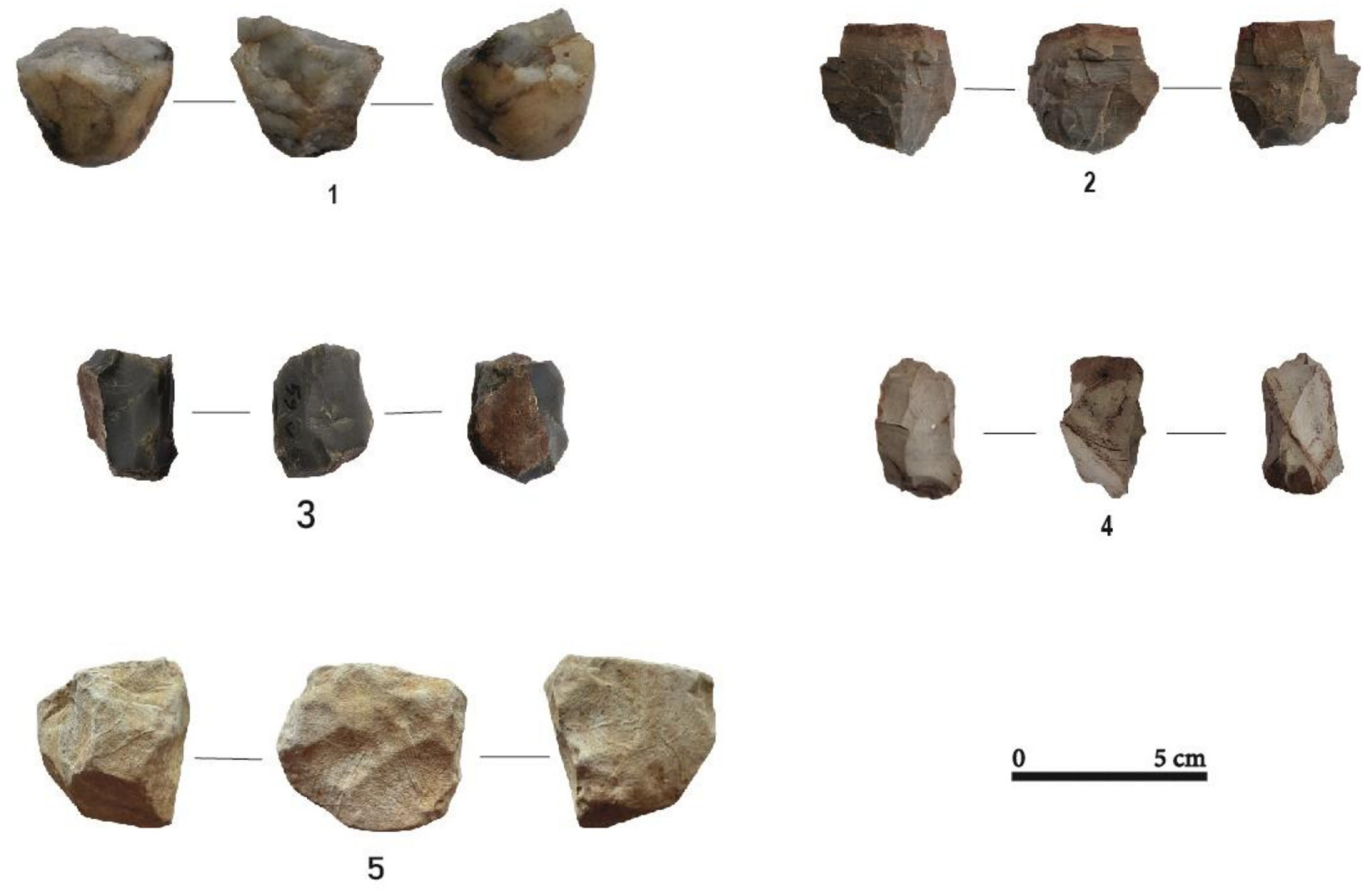
Different core exploitation modalities at Ravin of Sansandé. (1) Semi-rotating core, quartz. (2, 3) Frontal core, chert. (4) Peripheral core, chert. (5) Frontal core, greywacke.

The cores (n = 12) are made of chert (n = 6), quartz (n = 3) and greywacke (n = 3). The removal scars and the dimensions of these cores, indicate that their exploitation was directed towards the production of flakes, bladelets, blades and laminar flakes. Simple and opportunistic exploitation strategies were used, following different modalities (Table 10). In general, the core reduction for flake production is not standardized as reflected by the multiple orientations of the exploitation of nodules (unipolar, orthogonal, oblique, and bidirectional). Chert cores that were exploited frontally produced flakes and blades, while their exploitation in a semi-turned modality led to the production of bladelets only. Four cores on chert nodules have cortex over 1/3 of their surface. These cores are exploited frontally and abandoned in an initial stage, possibly due to the narrowness of the striking plane. The greywacke cores show only a frontal exploitation for the production of flakes according to the negatives of removal, although greywacke also produced a large proportion of blades and flakes. In fact, for three greywacke cores, frontal exploitation was used to obtain flakes (3), blades (2) and bladelets (1). This undoubtedly indicates the succession of several methods of exploitation on the same core (first for the blades and flakes, then for the bladelets) and that the *chaînes opératoires* on these materials are perhaps longer than for the other materials (Fig 10). In general, only one striking plane is used, and rarely two. The use of two striking planes seems to be related to the need to correct knapping errors or to rectify the convexities of the exploitation surface to allow the continuation of the reduction. The last removal scars visible on greywacke cores show slightly hinged terminations. Of the three quartz cores, one core shows flake removal scars while the other two shows flake and bladelet removal scars.

**Table 10:** Cores attributes in Ravin of Sansandé site. A) Dimensions of cores per raw material. B) Cores reduction modalities per cores main objectives and numbers of striking platforms.

**A.**		Length (mm)				Width (mm)				Thickness (mm)			
	N =	Min.	Max.	Mean	St.dev.	Min.	Max.	Mean	St.dev.	Min.	Max.	Mean	St.dev.
Chert	6	31	40	35	2.61	22	32	26	2.94	21	29	24	2.37
Quartz	3	33	42	36	3.87	24	31	27	2.88	15	24	18	3.87
Greywacke	3	36	45	41	3.66	35	43	38	3.36	29	36	32	2.88
**B.**	Cores with bladelet removals (n =)			Cores with flake removals (n =)		Cores with blade removals (n =)		Cores with mixed removal types (n =)		One striking platform (n =)		Two striking platforms (n =)	
Frontal	1			3		2		1		7			
Semi-rotating	3									2		1	
Peripheral	1			1						1		1	
Total	5			4		2		1		10		2	

#### 4.3.4. Flakes, blades and bladelets

**Fig 11:**
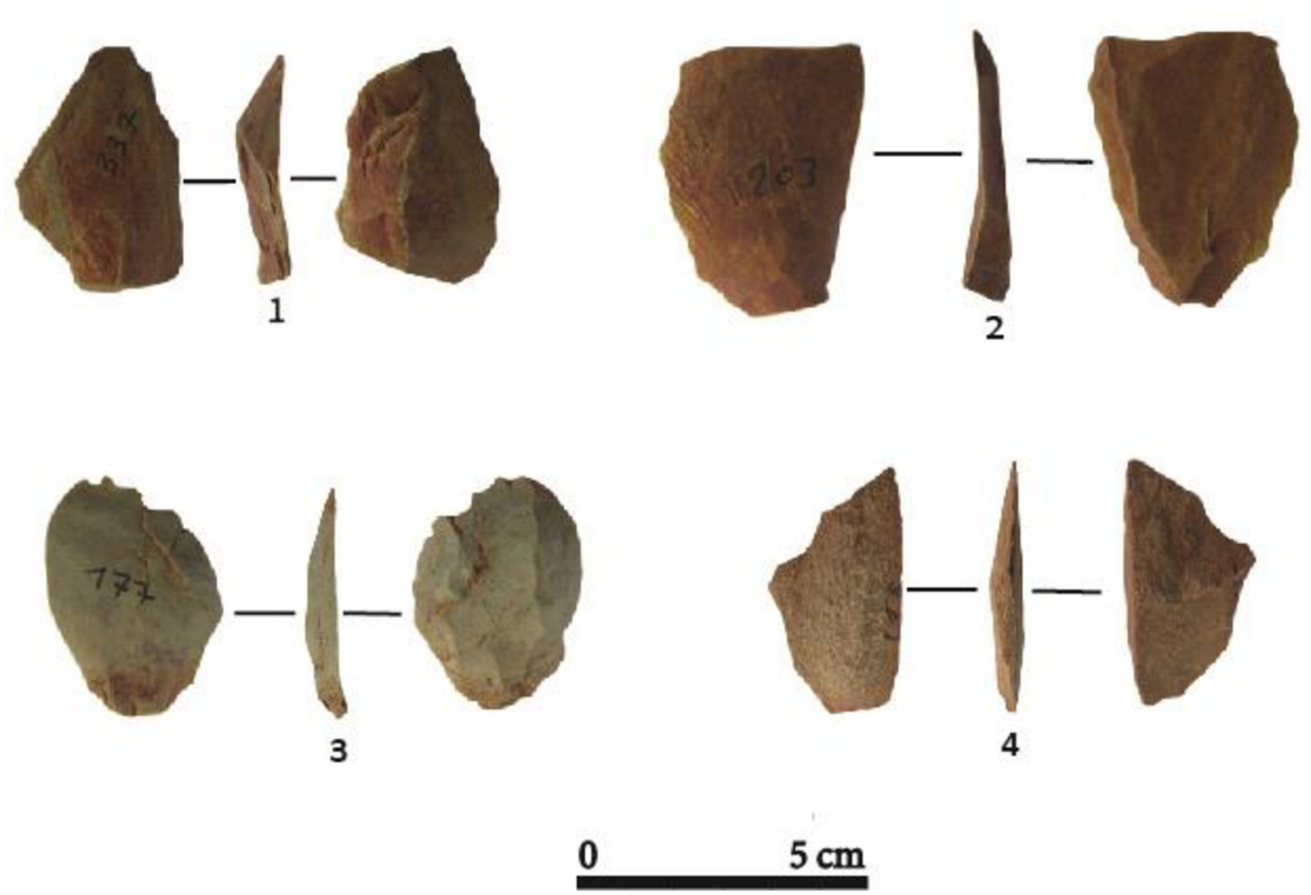
Flakes at the Ravin of Sansandé site. (1,3) flakes, chert. (2,4) Débordant flakes, greywacke.

The lithic assemblage includes a total of n = 396 diagnostic flakes (Fig 11; Table 11). These reflect, as the cores do, the exploitation of various raw materials, with a majority deriving from chert (67.17%) and a minority from quartz (5.8%). Flakes made of greywacke (27.03%) are also well represented, and show a great variability in their dimensions, as well as little standardization in their production. The profiles of the flakes are generally convex or concave (n = 249) and sometimes sinuous (n = 91) or twisted (n = 56). Semi-cortical flakes (n = 52) mirror the minimal preparation observed with the cores. The percentage and location of the cortex is extremely variable (semi-cortical, lateral, bilateral), but it is present on all the above-mentioned raw materials (Fig 11).

**Table 11:** Raw materials and dimensions of flakes, blades, bladelets, segments and end-scrapers of Ravin de Sansandé site.

			Length (mm)				Width (mm)				Thickness (mm)			
		N =	Min.	Max.	Mean	St.dev.	Min.	Max.	Mean	St.dev.	Min.	Max.	Mean	St.dev.
	Chert	266	29	50	31	1.66	20	42	24	1.12	2	25	11	1.01
Flakes	Quartz	23	34	59	37	4.62	13	34	28	3.36	3	20	12	2.5
N = 396	Greywacke	107	28	56	30	3.69	18	42	28	2.42	2	26	13	2.09
Blades N = 71	Chert	38	28	54	42	3.93	12	33	20	4	5	22	15	2.6
	Quartz	3	44	58	51	5.71	22	33	29	4.65	3	16	12	5.68
	Greywacke	30	28	52	39	4.55	12	27	18	2.88	4	14	14	2.98
Bladelets N = 83	Chert	32	21	37	27	2.06	8	12	10	0.5	4	15	10	1.37
	Quartz	5	22	30	26	2.52	10	12	10	0.89	4	15	7	3.82
	Greywacke	46	24	43	28	2.28	9	12	10	0.31	4	13	11	1.2
Segments N = 9	Chert	7	11	17	13	3.5	5	8	6	1.6	4	6	4	1.25
	Greywacke	2	15	18	16	1.5	7	9	8	1	4	5	4	1
End-scrapers N = 2	Chert	2	21	25	23	4	17	20	18	3	8	10	9	1
	Greywacke	1		22				16				6		

The flakes related to core production and maintenance (*débordant* flakes) show a debitage oriented towards the production of thick blanks, according to a simple and opportunistic exploitation of the cores. The *débordant* flakes (n = 78) show the removal of irregularities and the maintenance of the convexities of the cores’ exploitation surfaces. Cores with new striking platform and debitage surfaces produced a larger number of flakes (n = 243).

A few laminar flakes (n = 23) are found at the RDS site, but no cores present such flake scars. Laminar flakes can occasionally be produced from ovoid nodules whose surfaces are wide and short enough to accommodate this type of debitage. Blank production is generally carried out using dihedral platforms at the front of the exploitation surface, according to a simple process that consists of first in removing a few adjacent laminar flakes. Most flakes have peripheral cutting edges, which suggest that some flakes and laminar flakes are also desirable blanks.

**Fig 12:**
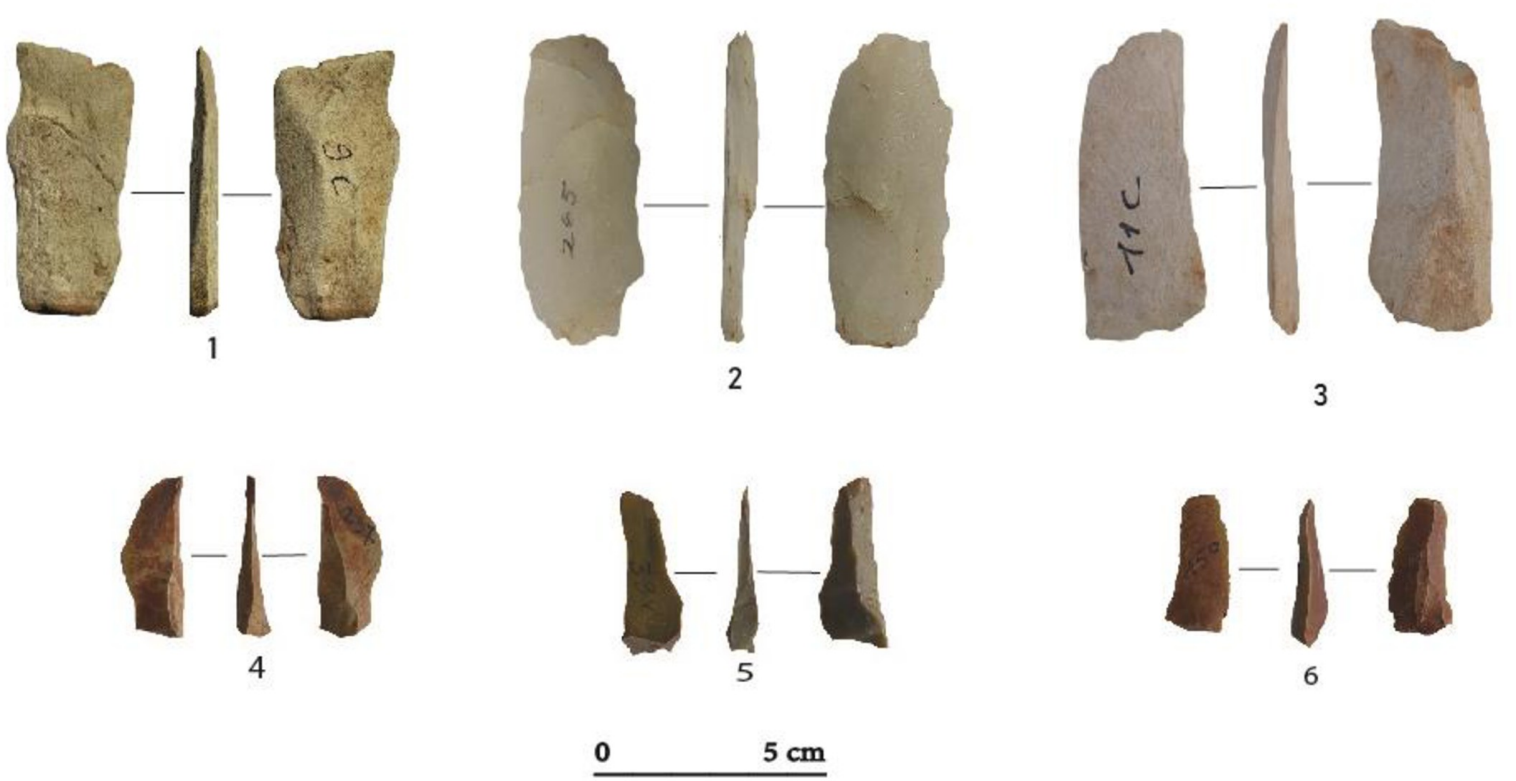
Blades and bladelets at the Ravin of Sansandé site. (1,3) blades, chert with patina. (2) blade, quartz. (4,6) bladelets, chert.

The n = 71 blades are relatively thick and wide (Table 11; Fig 12). Greywacke is over-represented in the class relative to its broader frequency in the assemblage (42.2% of all blades; Table 9). However, chert is the most frequent raw material (53.5%) while quartz is the least frequent (4.3%). They were produced on frontal cores, often exploited according to morphological opportunities offered by the convexities of the surfaces. Different core reduction phases have resulted in a small number of semi-cortical blades (n = 9), with residual cortical planes, most often located in the lateral or distal parts of the blades. Flank blades (n = 23) were produced during core maintenance. The blades, obtained by unipolar (n = 58) and orthogonal (n = 13) knapping on small cores, have convex or concave profiles (n = 64) and rarely sinuous profiles (n = 7). The platforms are generally plain (n = 49), sometimes facetted (n = 18), and rarely linear (n = 4). The blades are, therefore, not very standardized in terms of dimensions, platform types and profile morphologies, which seems to indicate a simple production scheme involving a brief laminar production process occurring just after the opening of a striking plane.

Bladelets are relatively numerous (n = 83) (Fig 12) and most often made on greywacke (55.4% of all bladelets). They were extracted from semi-rotating cores, although they can also be produced by the opportunistic exploitation of ridges naturally present on the nodules (Table 11). While most platforms are plain (n = 72), some are also punctiform (n = 6) or linear (n = 5). Bladelets can be quite narrow but also show morphological variability. Some of them (n = 24) are particularly short (< 20 mm long). It is possible that they represent unintended by-products produced during technical actions performed on the cores during their reduction.

#### 4.3.5. Tools

**Fig 13:**
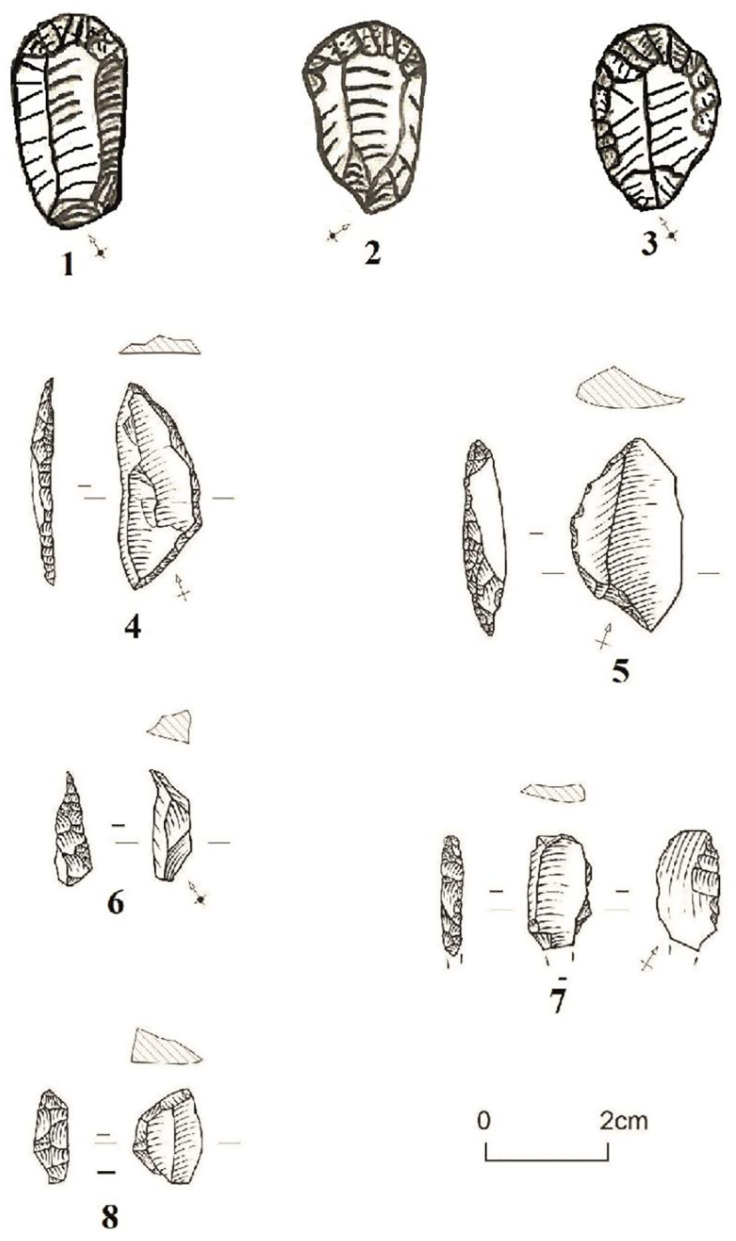
End-scrapers and Segments at the Ravin de Sansandé. (1,2) End-scrapers on a chert with rectangular and convex shapes with abrupt to semi-abrupt faces. (3) End-scraper on a greywacke with circular and convex shapes with abrupt to semi-abrupt faces. (4, 8) Segments with abrupt retouch and rectilinear edges. (5, 6) Segments with discontinuous retouch and irregular edges. (5, 8) Greywacke made on a flake. (4, 6, 7) Chert made on a bladelet.

The tool collection consists of nine segments and three end-scrapers. The nine segments are made on flakes (n = 4) and bladelets (n = 5) (Table 12). They are made of chert (n = 7) and greywacke (n = 2). The edges of six segments are convex with an abrupt retouch, opposite to a straight unretouched edge while the three others have discontinuous retouch and irregular edges (Fig 13).

**Table 12:** Segments at Ravin de Sansandé. Types and numbers of blanks used per raw materials.

Segments	Chert	greywacke	N =
Flakes	2	2	4
Bladelets	5		5
Total	7	2	9

Two of the end-scrapers are made of chert, while the other is made of greywacke. All three end-scrapers are made on flakes, and the morphology of their fronts is relatively similar, although two have rather rectangular shapes, while the third is more circular. The end-scrapers are small, 25 mm in maximum length, 20 mm in maximum width and 10 mm in maximum thickness and could, thus, be called microlithic. The profiles of the end-scrapers are convex with abrupt to semi-abrupt faces.

The analysis of the tools shows clear microlithism, with segments that are less than 20 mm in length. The three scrapers extend to a maximum of 25 mm in length. Within this microlithic geometrics are dominant. This interpretation is further supported by the presence of two end-scrapers with dimensions very close to those of the segments.

## 5. Discussion

The LSA industries from these two sites were found in the lower sedimentary units at TMBI-2017 (U_G_) and RDS (US1). Dating places these occupations at 16 ± 1 ka and between 13 ± 1 ka and 12 ±1.1 ka respectively, making them the only well stratified and dated Final Pleistocene Later Stone Age sites known so far in Senegal. The lithic assemblages show strong technological and typological similarities, suggesting a level of cultural affinity, as well as a substantial maintenance of technical traditions over more than three millennia. Geomorphological analyses show that both sites were occupied in a context of climatic improvement at the end of the Pleistocene and during the transition phase to the African Humid Period (14.8–5.5 ka) [53, 57]. The two sites presented here, found in stratified contexts, show similar technical and typological characteristics to numerous other LSA surface assemblages found in the area, as well as other recently excavated sites currently under study in the framework of our research program, “Human population and paleoenvironment in Africa”.

The evidence of substantial human occupation in the present-day Sudanian savanna during a period of increased moisture could indicate the attractiveness of the area and perhaps favorable conditions for the development of the new behaviors observed at these LSA sites [58–61]. These new behaviors can be seen in the transition from the flake-oriented industries of the MSA to the blades, bladelets, and microlithic industries of the LSA. However, the assemblages at TMBI-2017 and RDS suggest that the LSA was rooted in the long-term technical traditions of the populations along the Falémé River [42]. In parallel with a persistence of MSA technologies in the Senegal region [15–18, 62], the LSA, which is documented within a small number of sites in savanna zones, appears fully developed only after the Last Glacial Maximum, as conditions become wetter [22, 23, 63]. This possibility is even more interesting as parallel cultural trajectories can be found elsewhere in Senegal, where technical knowledge typical of the MSA technologies is still fully expressed until the Pleistocene-Holocene boundary [15–18, 59]. However, the LSA of the Falémé Valley differs from other assemblages attributed to the LSA in West Africa, mainly located farther south, in forested ecozones around the Gulf of Guinea [6, 8–10, 12, 13, 52, 62, 64, 65]. While investigating the mechanisms leading to the development of the LSA in the Falémé Valley is beyond the scope of this paper, we summarize below the main characteristics of these industries.

### 5.1. Summary on the Later Stone Age lithic assemblages from Toumboura I-2017 and Ravin de Sansandé

When comparing TMBI-2017 and RDS, the LSA industries are characterized by the use of varied and local raw materials, although in slightly different proportions. Actually it seems that quartz is well exploited at TMBI-2017 while greywacke is well exploited at RDS, and more than quartz, (mainly for blades and bladelets but also flakes). The same simple and opportunistic knapping strategies were employed at both sites, although the core reduction modalities are varied among each assemblage and according to the raw materials.

Flakes are slightly more frequent at RDS unlike TMBI-2017, where they are also more made from chert. The production of bladelets is higher at TMBI-2017 (23.3% of the total of flakes, blades and bladelets; RDS: 15.1%). Among bladelets, most are made on chert at TMBI-2017 (81.7%), which is in contrast to RDS (38.6% on chert), where they are mainly made on greywacke (55.4%). Blades are represented in similar proportions in both sites. However, at RDS great number of blades (n = 30) was made on greywacke while at TMBI-2017 greywacke blades are rare. At RDS chert blades are very numerous too, while quartz blades are poorly represented at both sites (that make another similarity between them). At RDS quartz flakes, blades and bladelets are poorly represented, while at TMBI-2017 quartz flakes are abundant. Quartz cores at TMBI-2017 are mainly exploited to produce flakes. The 39 segments (both on chert and quartz) are made either on flakes (n = 11), on bladelets (n = 24) or undetermined (n = 4). The 5 quartz segments are mainly on bladelets (n = 3) and rarely on flakes (n = 2). Conversely, at RDS greywacke is widely used (high proportion of greywacke flakes, blades and bladelets) together with chert, and much more than quartz.

The sites have in common the production of backed pieces of small dimensions, made on flakes and bladelets. Segments are the most numerous types of tools, with proportions varying from site to site (n = 39 at TMBI-2017 and n = 9 at RDS). These tools exhibit variable morphologies and are made from chert and quartz, but also from greywacke at RDS. They are distinguished, in most cases, by an abrupt, rarely irregular retouch, opposed to a straight unretouched edge. Besides the lower representation of segments at RDS, differences are identified with the presence of a few backed bladelets only at TMBI-2017 and end-scrapers only at RDS. The three end-scrapers are generally on convex flakes, with faces worked by marginal retouching. The very basic crafting of these end-scrapers argues in favor of their expedient character. The two backed bladelets show a short, direct, smooth retouch. The presence of these different types of tools and the different proportions of segments at each of the sites could be the result of different, activities carried out at the sites.

The two sites show similar types of cores reductions. At both sites, small nodules are prepared before main production phases, which follow simple and opportunistic management methods. The exploitation consisted of a short phase of cortex likely carried out of the sites and the search for one or rarely two cortical or smooth striking platforms, without specific orientation. The objectives of the production vary according to the raw materials. Chert cores are widely exploited in frontal or peripheral modalities for producing bladelets, flakes and blades. Cores on greywacke are reduced following a frontal modality, only for the exploitation of flakes and frontal quartz cores produced flakes and blades. Many blades and bladelets on greywacke are also produced in this material especially in Ravin de Sansandé. The semi-rotating modalities are widely favored on chert cores and sometimes on quartz for the production of bladelets. In general, the semi-rotating and peripheral modalities are aimed for the production of bladelets and rarely of flakes from two smooth striking platforms. Phases of maintenance of the convexities of the exploitation surfaces are also identified through flank blades and *débordant* flakes. The debitage sequences are in all cases short, producing few removals on the cores.

Each site shows several core reduction methods (e.g., frontal, semi-rotating, peripheral, opportunistic) and the use of different raw materials with a preference for chert at both sites and a representation of greywacke over quartz at RDS. Flake production is important at both sites and TMBI-2017 shows greater emphasis on the production of bladelets and segments. In general, the industry is small in dimensions, with average lengths ranging between 33 and 35 mm for the blanks (i.e., flakes, blades, and bladelets) and between 17 mm and 19 mm for the tools. Plain platforms are the most common for both sites. Due to the presence of both core preparation and core maintenance products, the knapping could have been carried out on site. These knapping activities may have been accompanied by other activities, requiring segments and backed bladelets at TMBI-2017 and segments and end-scrapers at RDS.

There are particularly few laminar flakes in the Ravin de Sansandé assemblage and no cores bear negatives of such removals. The presence of numerous chert flakes in both assemblages, despite its relative rarity in the local geology, shows that there was greater investment in the exploitation of this material than with quartz and greywackes. The production of blades and flakes, mostly with smooth ends and convex profiles, lend credence to the idea of a shared set of reduction practices at both sites by craftspeople sharing a common technical tradition.

### 5.2. Analysis and interpretation of the LSA assemblages from TMBI-2017 and RDS

The main features of LSA assemblages in the Falémé valley are production from small cores exploited in a frontal, semi-turned, or peripheral pattern. Nodules with removal negatives and cores were found in the stratigraphic units at both sites. There is a clear difference between the nodules with indeterminate removals, which are more like "geofacts" and, thus, excluded from this study, and the cores with frontal, semi-turned, and peripheral reduction, from which the artifacts analyzed in this article were knapped. However, it is difficult to say whether the cores were both prepared and reduced at the two sites, because the proportion of cortical blanks is low and no hammer stone were found. The availability of raw material sources near the sites suggests that the transport of previously decorticated cores cannot be excluded. However, further studies of the area’s raw materials and their relationships with the stratified sites will provide answers to this issue.

Local raw materials, deposited along the Falémé River, were exploited. High-quality (i.e. fine-grained, homogeneous, highly siliceous) raw materials, such as chert, are more highly reduced than lower-quality rocks such as quartz and greywacke. Relatively little effort has been invested in cores exploitation, probably due to the small initial size of the raw material. Small cores suggest relatively high rejection thresholds, as evidenced by nodules with small numbers of flake negatives cores excluded from the study. This could just as well reflect the preliminary work involved in preparing the before transporting them, or knapping them on site. The process of full debitage is generally carried out using a frontal and semi-turned exploitation, for efficient control of the core’s convexities.

Maintenance of the lateral convexity is also controlled by exploiting the periphery of the cores to extract *débordants* flakes or flank blades. Flaking surface maintenance is represented by *débordants* flakes or flank blades, which are related to the narrowness of the striking platform of the cores and, in particular, to the bending of the reduction surface. These maintenance flakes are generally linked to the appearance of asperities on the cores, which requires the extraction of *débordants* flakes or flank blades on the curved edges of the cores in order to open a striking platform. Attempts to restabilize reduction sequences at the striking platform can be challenging due to the reduction of the cores during the maintenance process, which will lead to core abandonment. The similarity of the reduction sequences, as well as the relatively small striking platform and debitage surface, show that simple, opportunistic exploitation was favored.

The low number of cortical flakes in all the assemblages suggests that the preparation of at least some cores took place outside the sites; whether this was near or far from the sources of raw materials remains unknown. Flakes, blades and bladelets are particularly frequent. In terms of quantity, flakes are the most abundant with the average dimensions of 29 mm in length, 23 mm in width, and 11 mm in thickness. Blades are of 9 mm thick, 21 mm wide and 41 mm length on average. A small proportion of laminar flake blanks, being longer on average (31 mm) than they are wide (24 mm), were found. The significant presence of flakes and bladelets at both sites may suggest that efforts were made to conserve the striking platform of the cores, in order to better optimize the detachment of these types of blanks from the debitage surfaces. The blades and bladelets of the two assemblages show very similar morphometric attributes, with the desired rectilinear profile suggesting a similar set of production techniques and goals at the two sites. Both the blades and bladelets are small and thin, with length/width ratio between 41mm / 21 mm for the blades and of 28 mm / 9 mm for the bladelets.

Tools are knapped on bladelets blanks (segments and backed bladelets) and flakes (end-scrapers and also segments). They are diverse in size, with no apparent morphological standardization. Analysis of the tools from the TMBI-2017 and RDS sites confirms the presence of identical dimensional groups, particularly within certain categories. The segment group appears homogeneous at TMBI-2017 in terms of dimensions, with average lengths of 14 mm, widths of 7 mm, and thicknesses of 4 mm. These same dimensions are present at RDS, where knappers produced 9 segments measuring 14 mm in length, 7 mm in width and 4 mm in thickness on average. Basically, the average dimension of the 2 backed bladelets is comprised between the average dimensions of the segments and end-scrapers. While the dimensional groups of the tools from the two sites show strong morphometric similarities for the segments, the consistency of the dimensions highlight the homogeneity for the backed bladelets and end-scrapers.

The microlithization of both assemblages is clearly attested by a wide range of flakes blanks and bladelets whose average dimensions are < 30–50 mm in length. The tools are also marked by the presence of small microlithic elements such as segments and backed bladelets, which characterize the LSA and make these sites rare in Senegal and West Africa for their stratified nature and associated absolute dates. This microlithic phenomenon observed at both sites can be considered as a form of continuity of the typo-technological objectives in the constitution of both lithic assemblages across millennia [54, 55]. However, we should not underestimate the complexity with which these microlithic assemblages relate to technical traditions and evolutionary strategies linked to ecosystem changes in time and space during the Final Pleistocene in West Africa [13, 66]. These analyses of the TMBI-2027 and RDS assemblages show a great deal of homogeneity. The production of blanks and tools shows high rates of dimensional convergence with TMBI-2017 and RDS flakes (≤ 30 mm on average). The average length of bladelets is 28 mm at TMBI-2017 and 30 mm for RDS. A possible alternative hypothesis for the low dimensional and behavioral variability of LSA lithic microlitization in the Falémé Valley is the process of convergent evolution structured by local raw materials and/or shared technical traditions.

Indeed, the emergence of LSA technology in the region seems to be structured by strategies for the exploitation and maintenance of small-sized cores through the preparation of the platform and debitage surfaces, as well as the production of flakes and bladelets at both sites. The adoption of microlithic technical behaviors at LSA sites can be seen as an intentional choice, encouraged by mobile production strategies, including the transport of materials and the multifunctional use of miniaturized blanks [55, 67, 68]. All these characteristics clearly point to Later Stone Age industries, and their similarities suggest a strong techno-cultural continuity in this part of the Falémé Valley between about 17 ka and 12 ka. These robust data are sufficiently consistent to reinforce the idea of the existence of a homogeneous local culture in Senegal that evolved during the Final Pleistocene within this broader West African technocomplex [19, 26, 69].

### 5.3. The LSA of the Falémé Valley in the context of the Final Pleistocene in West Africa

**Fig 14:**
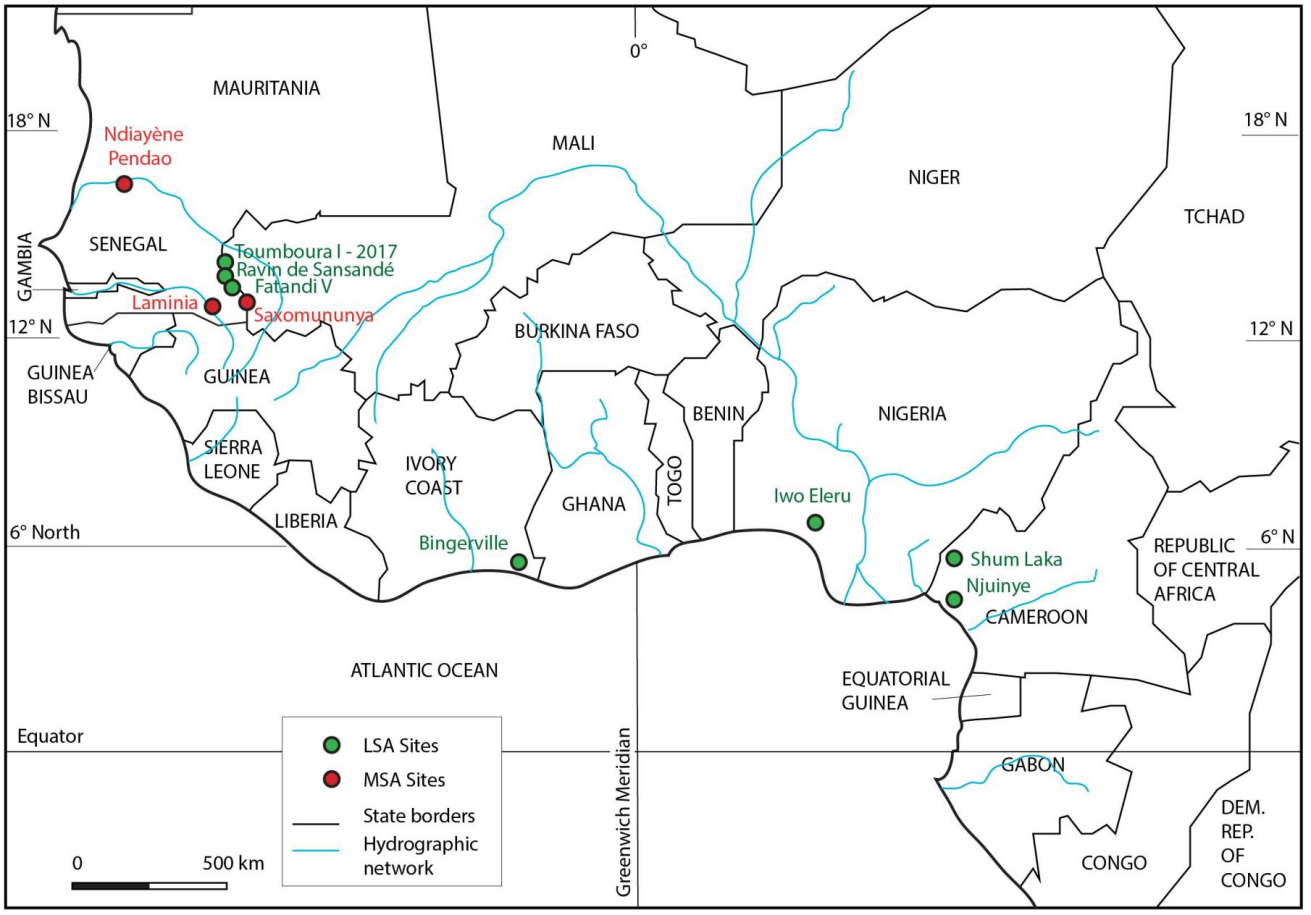
Location of Toumboura I-2017 and Ravin de Sansandé sites with MSA and LSA sites in West and Central Africa mentioned in the text.

Although Later Stone Age industries are abundant in Senegal, they are very rarely found in stratigraphic context, and this assessment can be extended to the whole of West Africa [22, 42, 70]. Beside the sites in the Falémé Valley, now including TMBI-2017 and RDS dating to the Pleistocene and Fatandi V dating to the Final Pleistocene/Holocene transition [22], the closest LSA stratified sites with good chronological control are located in the equatorial zone around the Gulf of Guinea (Fig 14; Table 13). This raises several questions–and many difficulties–regarding the interpretation of the population and techno-cultural dynamics at play during the beginning of the Later Stone Age in West Africa.

**Table 13:** OSL and C 14 dates from MSA and LSA sites cited in the article. We used OxCal to provide calibrated Before Present dates. The OSL ages from Scerri and al. 2017 and 2021 have been rounded to the first decimal. https://c14.arch.ox.ac.uk/oxcal/OxCal.html.*: OSL ages from Bayesian analyses, 95% credibility interval.

Sites	Layer	Dated sample ID	Uncalibrated date BP	Calibrated C14 ages (years, cal BP)	OSL age (ka)	Attributed cultural chronology	References
Njuinye	Layer 3	OS-21497	34.700 ± 560	41.130–38.570		LSA	Mercader and Martí, 2002
Njuinye	Layer 3	OS-22245	17.800 ± 180	22.085–21.017		LSA	Mercader and Martí, 2002
Shum Laka	S-deposit (Basal part)	OxA-4945	31.700 ± 750	38.406–34.591		LSA	Cornelissen, 2003
Shum Laka	S-deposit (Upper part)	OxA- 5200	12.800 ± 110	15.625–14.974		LSA	Cornelissen, 2003
Bingerville	1.3 m depth of the trench	Gif-5626	13.050 ± 230	16.345–14.976		LSA	Chenorkian et al. 1982
Iwo Eleru	Lowest Levels	I-1753	11.200 ± 200	13.452–12.756		LSA	Shaw and Daniels, 1984
Laminia	Depth 370 cm	Shfd-16115			22.0 ± 0.9 ka	MSA	Scerri et al. 2021
Laminia	Depth 370 cm	Shfd-16116			20.8 ± 0.8 ka	MSA	Scerri et al. 2021
Saxomununya	Depth 45 cm	Shfd-18020			11.1 ± 0.6 ka	MSA	Scerri et al. 2021
Ndiayène Pendao	Depth 28 cm	Shfd-15010			11.6 ± 0.5 ka	MSA	Scerri et al. 2017
Fatandi V	Layer 1	F10			12.8–9.6 ka*	LSA	Chevrier et al. 2020
Fatandi V	Layer 2	F12			14.3–10.3 ka*	LSA	Chevrier et al. 2020
Toumboura I-2017	Depth -170 cm	T7			16 ± 1 ka	LSA	
Ravin of Sansandé	Depth– 130 cm	S15			13.0 ± 1.0	LSA	
Ravin of Sansandé	Depth -130 cm	S16			12.0 ± 1.1	LSA	

The Fatandi V site provided ages very close to RDS, as it was dated to the Pleistocene/Holocene boundary between 12.8 and 10.3 ka [22]. The industry shows an almost exclusive selection of chert, contrary to TMBI-2017 and RDS, where quartz and greywacke also make up a significant part of the assemblages. Furthermore, at Fatandi V, the chert shows cortical surfaces of primary or sub-primary sources, whereas at TMBI-2017 and RDS, the cortex is rather alluvial or at least water-worn. Core reduction methods differ little between the three sites, with the use of simple methods and short exploitation sequences, producing a low number of blanks per cores; mostly bladelets and flakes. But at TMBI-2017 and RDS, cores reductions management can be bidirectional or orthogonal, in addition to the unidirectional knapping widely used at Fatandi V. The products are also smaller in size at TMBI-2017 and RDS compared to Fatandi V, which is clearly reflected in the tool category. All three sites have backed elements in their assemblages–mainly segments. However, notable differences exist. At Fatandi V, only two segments were found in stratigraphy. They are made of different materials (chert and quartz) from the rest of the assemblage and crafted on elongated products or bladelets of larger dimensions than those produced at the site. Their dimensions are >3cm, whereas at TMBI-2017 and RDS the segments are more numerous -at least in the oldest site-microlithic in size (<3cm). Consequently, there are both similarities (core reduction methods and tool categories) and differences (raw material management and dimensions) between these three LSA sites located in a restricted area along the Falémé river.

While these differences and similarities could be due to site function or technical flexibility as part of a culturally stable population, the hypothesis of partial cultural continuity combined with gradual adaptative changes over time cannot be ruled out. In both cases, all three sites are clearly anchored in the Later Stone Age, and do not seem to document a phase of emergence of this technocomplex.

Several Pleistocene sites with microlithic industries have been described around the Gulf of Guinea, at Bingerville in Ivory Coast and at Iwo Eleru, Nigeria (Fig 14). At Bingerville, an assemblage of n = 36 lithic artifacts were collected at a depth of 1.3 m in a roadwork trench and dated to 16.345–14.976 cal BP (Gif-5626: 13.050 ± 230 BP). The lithic industry consists of microlithic quartz flakes including scrapers, very small burins, a side scraper, and retouched flakes [4–6].

The Iho Eleru site revealed in its lower stratigraphic unit, lithic material including geometric microliths dated between 13.452–12.756 cal BP/ (I. 1753: 11.200 ± 200 BP) [8, 28]. The LSA lithic assemblages from these two sites, located in forest zones, present a microlithic technology exclusively based on quartz. This appears all the more interesting if one considers that in Sahelian contexts, the sites of Toumboura I-2017 and Ravin de Sansandé have rather developed technical behaviors favoring a variety of siliceous rocks, whose properties are different. Microlithic on quartz dominate at savanna sites, in contrast to forest sites where microlithic artifacts are rare, for example at Iho Eleru, Shum Laka and Bingerville [6, 9, 10, 28]. The choice of a quartz lithic industry may be linked to its availability as compared to other raw materials in the area. But this hypothesis, using an environmental factor as the determinant for the choice of raw material, would require further research before any confirmation. In the Sahelian zone, quartz in association with raw materials such as chert and greywacke has been collected in the lower levels of TMBI-2017, RDS, and Fatandi V in Senegal. The microlithic of the latter three sites, mainly made up of chert, greywacke and quartz, share some similarities with those of Iho Eleru, but are distinct from the quartz tools (e.g., scrapers, end-scrapers, burins and retouched flakes) of the Bingerville site. The specific reasons for the use of quartz in these areas seem to be dictated by the better visibility of these types of raw materials in the savanna landscape context. However, in forest sites where vegetation conditions are rather dense, access to more durable raw materials available in larger nodules, such as greywacke and chert, is more suitable for the production of lithic blanks or tools [40, 71, 72]. However, it is difficult to rule out the possibility that local populations, with technical knowledge and behavior acquired over a long period of time, would enable them to make choices about one or more raw materials (quartz only, for example) according to the conditions offered by the environmental context in which they lived [73]. While at the Bingerville sites, microlithic tooling is expressed mainly through quartz scrapers, burins and retouched flakes, at Toumboura I-2017, and at Ravin de Sansandé, these tool types are absent. Instead, segments, backed bladelets and end-scrapers on a variety of raw materials are found. Thus, these sites suggest a contemporaneous evolution of LSA technical behavior in relatively different ecosystems. Indeed, the Bingerville site is dated to 16.345–14.976 cal BP at an age close to that of TMBI-2017 (17–16 ka), while the RDS site (13–12 ka) is similarly dated to Iho Eleru (13.452–12.756 cal BP).

LSA stratigraphic sequences have also been identified in Central Africa at Njuinye and Shum Laka in southwestern and northern Cameroon respectively [7, 9, 10, 13, 74]. However, the Central African LSA presents few sites with a wide diversity of industries associated with reliable stratigraphic and chronological contexts [34]. The LSA in this region is not homogeneous, which requires us to be careful when comparing these material cultures to those of Senegal. The data from Central Africa do however give us an overall view of the evolution, diversity, and complexity of LSA technology in both tropical forest and savanna areas. In Layer 3 of the Njuinye site, dated to 41.130–38.170 cal BP, an LSA-type quartz industry appears, consisting of a few geometric pieces, drills, large wide scrapers, notches, and denticulates [11]. In the lower part of the Shum Laka sequence, dated c.30-12ka to 38.406–34.591 cal BP / OxA-4945: 31.700 ± 750 BP) and 15.625–14.974 cal BP / OxA-5200:12.800 ± 110), quartz industries are also developed, mostly composed of small flakes, few geometric microliths and non-geometric microlithic tools [7, 9, 10, 13]. Artifacts from these two sites show some similarity in microlithic tools to the sites of Toumboura I-2017 and Ravin de Sansandé in the Falémé Valley, although typical LSA formal tools such as segments and backed bladelets are not common in the Cameroonian case. The major difference is a greater variety of raw materials selected at the LSA sites in Senegal, at a time when a non-standardized microlithic technology, primarily on quartz, with no change in raw material supply or technology, is present over the long-term at Njuinye and Shum Laka.

Thus, the LSA assemblages from Toumboura I-2017 and Ravin de Sansandé appear to be a unique local variant in West Africa, with typical technological characteristics and without equivalent. Beyond the microlithic tools, they present some typological differences between the LSA assemblages of West and Central Africa covering the period from 40–21 ka at Njuinye and Shum Laka to ca. 16–12 ka at Bingerville and Iwo Eleru. The production of microlithic tools over a long period of time and on a large local and regional scale may indicate a significant degree of technical and environmental adaptive flexibility and demographic stability of population groups present in northwest and central Africa [27, 75]. It remains difficult to assess the influence of environment on the diversity and differences of LSA industries in West and West-Central Africa since the food resources exploited are not always known [7, 23, 76]. The technical and transport flexibility offered by the production of microlithic blanks certainly facilitated the local emergence and diffusion of these tool types around 17–16 ka at TMBI-2017, and then maintained despite the ecosystem transformations that mark the end of the Pleistocene and the transition to the Holocene at around 13–12 ka at Ravin de Sansandé (Table 10).

The Later Stone Age appears in a context that is also contemporary with groups still fully employing Middle Stone Age technologies with Levallois and discoid production, until at least the Final Pleistocene/Holocene transition. In the upper part of Unit 1B at the site of Laminia, located on the Gambia River terraces in southeastern Senegal, dated between 22.0 ± 0.9 ka (Shfd16115) and 20.8 ± 0.83 ka (Shfd16116), an MSA assemblage composed of Levallois cores, bifacially retouched flakes, and laterally retouched flakes and scrapers was collected [18]. At the Ndiayène Pendao site located on the Senegal River terraces, the MSA, featuring classic MSA core axes, basally thinned flakes, Levallois points and denticulates mostly made from chert was dated to 11.6 ± 0.5 ka [16–18]. However, the discovery of an MSA industry at the Saxomununya site, on a fluvial terrace surface on the left bank of the Falémé River is particularly interesting, given the proximity to the Ravin de Sansandé, Toumboura I-2017 and Fatandi V sites. The Saxomununya site is dated to 11.1 ± 0.58 ka, which is younger than the Ravin de Sansandé and Toumboura I-2017 sites and possibly equivalent in age to Fatandi V. The MSA industry collected between 40–50 cm of depth at the Saxomununya site is dominated by Levallois cores, discoidal cores for the production of flakes, and a classic MSA tool types, including denticulates, side and end scrapers, notches, retouched Levallois flakes, and foliate [18]. Taken together, the evidence for the late survival of MSA technology at Laminia, Saxomununya and Ndiayène Pendao at a time when LSA assemblages predominate in West Africa suggests the possibility that two distinct population groups practiced relatively contrasting technological strategies in similar spatio-temporal and environmental contexts, at least within the vicinity of the rivers of northern and eastern Senegal [15–18].

The LSA sites of Toumboura I-2017 and Ravin de Sansandé in the Falémé Valley provide new data that allow us to place West Africa in the debates on the diffusion of anatomically modern humans in relation to the technical behaviors at work on the African continent [12, 57, 77, 78]. Recent genetic studies based on modern DNA show that modern humans in West Africa underwent introgression towards the end of the Middle Pleistocene, from a group related to archaic populations [73, 79–81]. These ancient genome transfers align with paleoanthropological data from Iho Eleru in southwestern Nigeria where the oldest known human fossil from West Africa exhibits certain archaic anatomical features [12, 62, 64, 77]. The Later Stone Age sites in eastern Senegal, like their contemporary neighbor Iho Eleru, appear to belong to anatomically modern population groups that were able to adapt to variations in the region’s ecosystems thanks to hospitable refuges offered along the banks of the Falémé River. The techno-typological continuities or breaks between the Later Stone Age assemblages of West Africa may reflect the isolation, persistence, and periodic dispersal of *Homo* sapiens populations in this region during the Final Pleistocene.

## 6. Conclusion

The sites of Toumboura I-2017 and Ravin de Sansandé in the Falémé Valley provide new data on Later Stone Age technological behaviors and adaptive strategies of modern humans in relation to environmental changes observed during the Final Pleistocene to early Holocene in West Africa. Lithic assemblages collected from the archaeological levels of both sites combined with stratigraphic data and OSL dating, suggest that human were present in eastern Senegal at least between 17–16 ± 1 ka at Toumboura I-2017 and 13.0–12.0 ± 1.0 ka at Ravin de Sansandé as climatic conditions became wetter [53] during the local initiation of the African Humid Optimum. The study of the *chaîne opératoire* of the lithic assemblages revealed the use of diverse local raw materials based on simple and opportunistic exploitation patterns, for the production of flakes, blades and bladelets that were not standardized. The cores of both assemblages were all minimally prepared and reduced–sometimes to exhaustion–in frontal, semi-rotating and peripheral modalities on small nodules. The tooling is limited to the transformation of flakes and bladelets, mainly into microlithic segments, but also into end-scrapers and backed bladelets. The results obtained attest to the oldest known LSA sites in Senegal, contemporary with the Iho Eleru site, which yielded the only Stone Age *Homo sapiens* fossil in West Africa. Comparisons of lithic production from LSA sites of the Falémé valley, at Toumboura I-2017 and Ravin de Sansandé, but also at Fatandi V, with other LSA assemblages documented in West and Central Africa at Bingerville, Iho Eleru, Njuinye, and Shum Laka, suggest the existence of relatively different technical behaviors in spatio-temporal and environmental contexts that are sometimes contrasting. Thus, the hypothesis of a local variability of LSA technological behaviors in West Africa seems likely.

## Supporting information

S1 File (DOCX)
