# Supplementary material for: Two new Later Stone Age sites from the Final Pleistocene in the Falémé Valley, eastern Senegal
Source: PLoS One. 2024 Mar 28;19(3):e0294346. doi: 10.1371/journal.pone.0294346 (PMC10977785; doi:10.1371/journal.pone.0294346)
Supplement: S1 File — (DOCX) [file pone.0294346.s001.docx]

**Two new Later Stone Age sites from the Final Pleistocene: Toumboura I-2017 and Ravin de Sansandé in the Falémé valley, eastern Senegal.**

**Supplementary information for the Optical dating.**

1. **Equivalent dose**

1-1) Measurements devices

Measurements were performed on a Lexsyg Smart reader for multi-grain stimulation, and a Risoe TLDA 20 reader for single grain stimulations (Richter et al., 2015, Botter-Jensen et al., 2003). The Lexsyg Smart is equipped with green LEDs (525 nm) for stimulation, and a Hamamatsu H7360-02 photomultiplier tube preceded by 2.5 mm Hoya U340 and Delta BP 365/50 EX filter for detection. The Risoe reader is equipped with a 10 mW Nd: YVO4 diode- pumped laser (532 nm) for stimulation and a PDM 9107Q-AP-TTL-02 photo-multiplier tube preceded by 7 mm Hoya U340 filter (range 280-380 nm) for detection. A ^90^Sr/^90^Y beta source is attached to each reader, delivering respectively 0.15 Gy/ s and 0.10 Gy/s to the quartz grains.

1-2) Tests

1-2-1) LM-OSL test

Linearly Modulated luminescence measurements were performed on one multi-grain aliquot for each sample. The green stimulation was increased from 0 to 70 mW/cm^2^ in 1000 s at 125°C after a preheat at 260°C for 10 s. The Risoe calibration quartz (Hansen et al., 2015), known to be dominated by the fast component was used as a reference. On figure sup 1 a and b, the LM-OSL curves for the natural signals are shown.

It can be observed that the signal is dominated by the fast component for all samples. Consequently, the Single Aliquot and Regenerative dose protocol has been tested and applied.


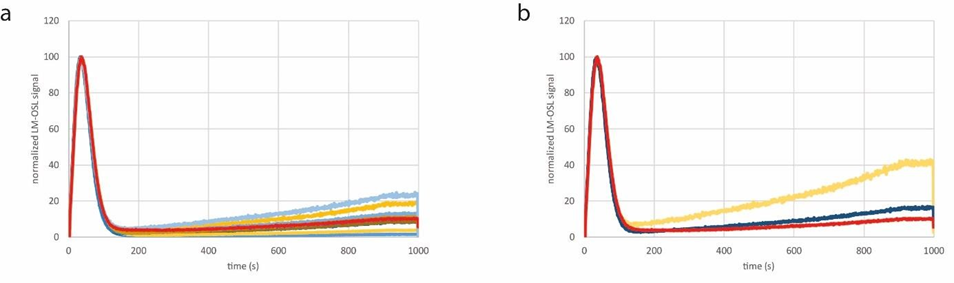


**Fig 1. LM-OSL measurements.** All measurements (one per sample) have been normalized. The red curve in both figures corresponds to the Risoe calibration quartz. a) measurements for T1 to T9: b) measurements for S15 and S16.

1-2-2) Dose recovery tests

Each sample was bleached for one minute in a solar simulator (Hönle UVAcube 400), and bleached again in the reader (blue LED for 200 s at room temperature) after a pause of at least 10000 s. A beta dose, close to the expected equivalent dose (De), was given in the reader, and the Single Aliquot and Regenerative dose protocol (SAR, Murray and Wintle, 2000) was performed (**Fig 2**). Preheat of 260°C for 10s for the natural and regenerative doses and cutheat of 160°C for the test doses were applied. Results are summarized in Table 1. They are all consistent with unity. Consequently, the same preheat and measurement conditions have been applied to the natural samples.

|  |  |
| --- | --- |
| irradiation (a) | |
|  |  |
| preheat 260°C 10s (b) | |
|  |  |
| OSL @125°C for 0.85s -> Lx (c) | |
|  |  |
| irradiation (d) | |
|  |  |
| cutheat 160°C | |
|  |  |
| OSL @125°C for 1s -> Tx (c) | |
|  |  |

|  |  |
| --- | --- |
| irradiation (a) | |
|  |  |
| preheat 260°C 10s (b) | |
|  |  |
| OSL @125°C for 0.85s -> Lx (c) | |
|  |  |
| irradiation (d) | |
|  |  |
| cutheat 160°C | |
|  |  |
| OSL @125°C for 1s -> Tx (c) | |
|  |  |

repeat with doses D, 2D, 4D, 8D, 0, D

**Fig 2. SAR protocol.** a) No irradiation was performed for the first cycle of the natural equivalent dose measurements. b) Temperature was increased at 5°C/s. c) Signals Lx and Tx were integrated on the first 0.06 s and background for the last 0.12 s. d) Test dose was 5.5 Gy (S15, S16) or 7 Gy (T1 to T9).

| sample | N | selected | dose to recover (Gy) | dose recovery ratio | | OD | |
| --- | --- | --- | --- | --- | --- | --- | --- |
| T1 | 100 | 12 | 55 | 1.00 | ± 0.02 | 0 |  |
| T2 | 300 | 24 | 32 | 1.03 | ± 0.02 | 4 | ± 3 |
| T3 | 300 | 23 | 45 | 1.01 | ± 0.03 | 5 | ± 4 |
| T4 | 300 | 18 | 32 | 1.01 | ± 0.02 | 0 |  |
| T5 | 300 | 24 | 32 | 0.98 | ± 0.02 | 6 | ± 3 |
| T6 | 300 | 24 | 45 | 0.97 | ± 0.02 | 0 |  |
| T7 | 300 | 20 | 23 | 1.03 | ± 0.02 | 0 |  |
| T8 | 300 | 23 | 23 | 1.03 | ± 0.02 | 0 |  |
| T9 | 300 | 20 | 22 | 1.01 | ± 0.02 | 0 |  |
| S15 | 300 | 26 | 20 | 1.00 | ± 0.02 | 0 |  |
| S16 | 300 | 18 | 20 | 1.00 | ± 0.02 | 0 |  |

**Table 1**. **Results of the dose recovery test**. N: number of measured grains. Selected: number of grains that pass the selection criteria (see main text). Dose recovery ratio: ratio between the estimated central dose (central age model, Galbraith et al., 1999) and the dose to recover. OD: overdispersion. Note here that they are all consistent with 0 (for T5 at two sigma); i.e. there is no significant overdispersion.

1-3) De determination

Fig. 2 and Table 2 summarize the results for the equivalent dose determination.

| sample | N | pass criteria | Equivalent Dose CAM (Gy) | | OD (%) | | Equivalent Dose FMM (Gy) | |
| --- | --- | --- | --- | --- | --- | --- | --- | --- |
| T1 | 1200 | 166 | 44.8 | ± 1.1 | 29 | ± 2 |  |  |
| T2 | 1200 | 63 | 28.3 | ± 0.9 | 23 | ± 3 |  |  |
| T3 | 1200 | 75 | 37.3 | ± 1.6 | 34 | ± 3 |  |  |
| T4 | 1200 | 58 | 35.5 | ± 1.6 | 31 | ± 3 |  |  |
| T5 | 1200 | 77 | 39.7 | ± 1.7 | 34 | ± 3 |  |  |
| T6 | 1100 | 87 | 37.3 | ± 2.1 | 50 | ± 4 | 38.9 | ± 1.4 |
| T7 | 1200 | 85 | 34.5 | ± 2.3 | 59 | ± 5 | 38.0 | ± 1.4 |
| T8 | 1200 | 106 | 21.7 | ± 1.4 | 62 | ± 5 |  |  |
| T9 | 1200 | 84 | 6.1 | ± 0.6 | 96 | ± 8 |  |  |
| S15 | 1200 | 129 | 19.0 | ± 1.0 | 61 | ± 4 | 22.9 | ± 0.7 |
| S16 | 1200 | 87 | 17.6 | ± 1.2 | 63 | ± 5 | 20.5 | ± 0.8 |

**Table 2. Equivalent doses data**. N: number of measured grains. Pass criteria: number of selected grains. CAM: central Age Model; OD: overdispersion; FMM: Finite Mixture Model. Only the main component is indicated. For these four samples, it gathers over 90% of the grains.

The overdispersion for samples S15 and S16 is rather high (respectively 61±4 and 63±5%). The radial plots of S15 and S16 suggests that at least two populations are present in the De distributions. Observations at the macroscopic scale in the field suggest possible bioturbations. Therefore, the Finite Mixture Model (Roberts et al., 2000) was applied to these samples. Sigma_b (overdispersion due to measurements and beta dose rate heterogeneities) was increased from 20 to 40% and the best fit and number of component were deduced from Bayes Information Criteria (BIC) and Maximum likelihood. In most cases a major component close to 20 Gy was obtained, i.e. the value of sigma_b has little influence on the De of the main component for these samples. The De reported on Table 2 corresponds to the De obtained for sigma_b of 30%.

For samples T1 to T5, the overdispersions stand between 29±2 and 34±3% and no particular pattern in the distribution plots is observed. The Central Age Model (Galbraith et al., 1999) was applied. For samples T6 to T9, the overdispersions are significantly higher than for the lower samples, from 50±4 to 96±8 %. Presence of a few isolated grains with either very high (e.g. >70 Gy) or very low (e.g. close to 0 Gy) dose can be observed. T6 to T9 correspond to a change in the sedimentation compare to the previous samples: the colluvial-alluvial system is initiated in a floodplain that is seasonally very wet at the beginning, with increased potential for poor bleaching, mixing of sediments and decreasing aggradation rate. Consequently, the Finite Mixture Model was applied. While a dominant component close to 40 Gy could be deduced for T6 and T7, it was not the case for T8 and T9, i.e. the Finite Mixture Model does not allow to calculate reliable ages for these two samples.

1. **Dose rate**

2-1) activities

Activities of the 238U and 232Th series or K contents have been determined by High Resolution Gamma Spectrometry at the IRAMAT-CRP2A (e.g. Guibert et al., 2009). Results are presented on Table supX2. For the U series, small (<30%) disequilibria can be observed between the head (pre-226Ra) and middle (post- 226Ra) or middle and bottom (210Pb) parts of the chain. The causes of these disequilibria are not precisely known. We assumed that theu had only a minor impact on the total dose rate, since U contributes for ca 20-30% to the total dose rate.

The activities and content are variable along the Toumboura 1 section. T2 and T3 in particular display much lower contents (e.g. 0.45 and 0.50 % K instead of about 1% for the other samples). This might be correlated to the grain size composition of the corresponding layers. T2 in particular is much sandier (60% sand after granulometric laser analyses), than the other samples.

For the Sansandé samples, S15 and S16, the two series of contents/activities are remarkably consistent, while both samples are 14 m distant, but supposed to come from the same sedimentary unit, suggesting that this one is homogeneous at the meter scale.

| Sample | activities (Bq/kg) | | | | | | | | content (%) | |
| --- | --- | --- | --- | --- | --- | --- | --- | --- | --- | --- |
|  | ^238^ U series | | | | | | ^232^Th series | | K | |
|  | top | | middle | | bottom | |  |  |  |  |
| T1 | 40.7 | ± 2.3 | 30.8 | ± 0.5 | 34.5 | ± 3.3 | 45.2 | ± 0.6 | 1.04 | ± 0.03 |
| T2 | 24.2 | ± 1.6 | 19.9 | ± 0.4 | 27.9 | ± 2.6 | 22.9 | ± 0.4 | 0.45 | ± 0.02 |
| T3 | 26.4 | ± 1.7 | 21.8 | ± 0.4 | 30.2 | ± 2.8 | 25.2 | ± 0.4 | 0.50 | ± 0.02 |
| T4 | 29.8 | ± 2.4 | 31.6 | ± 0.6 | 29.3 | ± 3.4 | 38.9 | ± 0.6 | 1.11 | ± 0.03 |
| T5 | 35.1 | ± 2.1 | 33.3 | ± 0.5 | 31.2 | ± 3.0 | 38.8 | ± 0.5 | 1.06 | ± 0.03 |
| T6 | 36.7 | ± 1.9 | 36.7 | ± 0.5 | 40.3 | ± 3.0 | 40.5 | ± 0.5 | 1.14 | ± 0.02 |
| T7 | 39.7 | ± 2.1 | 41.4 | ± 0.6 | 34.4 | ± 3.0 | 44.8 | ± 0.5 | 1.11 | ± 0.02 |
| T8 | 42.4 | ± 1.8 | 30.4 | ± 0.4 | 29.0 | ± 2.5 | 42.4 | ± 0.4 | 1.01 | ± 0.02 |
| T9 | 33.0 | ± 1.6 | 26.0 | ± 0.4 | 27.1 | ± 2.2 | 35.0 | ± 0.4 | 0.90 | ± 0.02 |
| S15 | 27.4 | ± 1.9 | 26.8 | ± 0.4 | 21.6 | ± 3.3 | 35.6 | ± 0.4 | 0.74 | ± 0.01 |
| S16 | 27.2 | ± 1.9 | 23.1 | ± 0.3 | 19.7 | ± 3.2 | 33.5 | ± 0.4 | 0.67 | ± 0.01 |

**Table 2.** 238U series and 232 Th series activities or K content for the samples from Toumboura 1 and Sansandé. They were determined by High Resolution Gamma Spectrometry. For the U chain, the top activity was deduced mainly from the ^234^Th gamma rays, the middle from ^214^ Bi and ^214^Pb, and the bottom from the ^210^Pb.

2-2) Total dose rates and Ages

A summary for the dose rate data, equivalent dose data and ages are given in Table 4 and sup 6.

| sample | grain size  (µm) | water  content (%) | burial depth  (m) | dose rate (Gy/ka) | | | | | | | |
| --- | --- | --- | --- | --- | --- | --- | --- | --- | --- | --- | --- |
|  |  |  |  | beta | | gamma | | cosmic | | total |  |
| T1 | 100-140 | 6% | 6.3 | 1.19 | 0.10 | 0.83 | 0.05 | 0.09 | 0.01 | 2.11 | 0.11 |
| T2 | 100-140 | 1% | 4.8 | 0.63 | 0.04 | 0.59 | 0.04 | 0.11 | 0.01 | 1.32 | 0.06 |
| T3 | 100-140 | 3% | 3.8 | 0.65 | 0.05 | 0.74 | 0.05 | 0.12 | 0.01 | 1.52 | 0.07 |
| T4 | 100-140 | 7% | 3.5 | 1.18 | 0.11 | 0.80 | 0.05 | 0.13 | 0.01 | 2.11 | 0.12 |
| T5 | 100-140 | 7% | 3.1 | 1.17 | 0.10 | 0.80 | 0.05 | 0.13 | 0.01 | 2.10 | 0.11 |
| T6 | 100-140 | 7% | 1.9 | 1.25 | 0.11 | 0.89 | 0.06 | 0.15 | 0.01 | 2.29 | 0.12 |
| T7 | 100-140 | 7% | 1.7 | 1.29 | 0.11 | 0.88 | 0.06 | 0.16 | 0.01 | 2.32 | 0.12 |
| T8 | 100-140 | 7% | 1.0 | 1.17 | 0.10 | 0.84 | 0.05 | 0.17 | 0.01 | 2.18 | 0.11 |
| T9 | 100-140 | 5% | 0.7 | 1.01 | 0.09 | 0.72 | 0.05 | 0.18 | 0.01 | 1.91 | 0.10 |
| S15 | 200-250 | 9% | 0.9 | 0.84 | 0.08 | 0.74 | 0.08 | 0.18 | 0.02 | 1.76 | 0.11 |
| S16 | 100-140 | 17% | 0.9 | 0.81 | 0.08 | 0.72 | 0.08 | 0.18 | 0.02 | 1.71 | 0.11 |

**Table 4.** Data for the calculation of the dose rate. The water content corresponds to what was measured at the time of sampling (from the back of the hole drilled for the insertion of the field gamma spectrometer probe). Beta dose rate is calculated from the U, Th, K content of each sample (from High Resolution Gamma

Spectrometry); gamma dose rate was determined with a field gamma spectrometer. Cosmic dose rate was based on the Prescott and Hutton (1994) equation, taking into account the burial depth. Uncertainties include both statistical and systematic uncertainties.Note that these results slightly differ from those of Lebrun et al. (2016) and Lebrun (2018), for T1 to T9, mainly because those were based on multigrain measurements instead of single grains measurements and calibrations of the sources have been improved.

T3 and T5 are slightly too old compare to the other samples, though the chronostratigraphic reversals are not statistically significant. Ages for Toumboura 1 are contemporaneous to MIS

2. The two ages for Sansandé that come from the same sedimentary unit are statistically consistent at 13.0+-1.0 and 12.0+-1.0 ka, at the very end of the Pleistocene.

| sample | Equivalent dose (Gy) | | Dose rate (Gy/ka) | | Age (ka) | |
| --- | --- | --- | --- | --- | --- | --- |
| T1 | 44.8 | ± 1.1 | 2.11 | ± 0.11 | 21 | ± 2 |
| T2 | 28.3 | ± 0.9 | 1.32 | ± 0.06 | 21 | ± 1 |
| T3 | 37.3 | ± 1.6 | 1.52 | ± 0.07 | 25 | ± 2 |
| T4 | 35.5 | ± 1.6 | 2.11 | ± 0.12 | 17 | ± 1 |
| T5 | 39.7 | ± 1.7 | 2.10 | ± 0.11 | 19 | ± 1 |
| T6 | 38.9 | ± 1.4 | 2.29 | ± 0.12 | 17 | ± 1 |
| T7 | 38.0 | ± 1.4 | 2.32 | ± 0.12 | 16 | ± 1 |
| T8 |  |  | 2.18 | ± 0.11 |  |  |
| T9 |  |  | 1.91 | ± 0.10 |  |  |
| S15 | 22.9 | ± 0.7 | 1.76 | ± 0.11 | 13.0 | 1.1 |
| S16 | 20.5 | ± 0.8 | 1.71 | ± 0.11 | 12.0 | 1.0 |

**Table 5**. Final equivalent doses, dose rates and ages for the samples for Toumboura 1 and Sansandé. The uncertainties for the ages include a 4% systematic uncertainty for the calibration of the beta source attached to the OSL reader.

# References

Bøtter-Jensen, L., Andersen, C.E., Duller, G.A.T., Murray, A.S. [Developments in radiation,](http://ehis.ebscohost.com.gate3.inist.fr/eds/viewarticle?data=dGJyMPPp44rp2%2fdV0%2bnjisfk5Ie46bFMsq6wTbakxmus2%2bKLvq6tSbelsEivqZ5QuKqzUrCmnlnLnPKK3%2bTxeeHq54fs3%2bJVq6euTbOmsFGyq7M%2b6tfsf7vc8D7i2Lt94unjhO6c8nnls79mpNfsVdGnsU2uqrJQtaaxSK6msEi3nOSH8OPfjLvc84Tq6uOQ8gAA&hid=104) [stimulation and observation facilities in luminescence measurements.](http://ehis.ebscohost.com.gate3.inist.fr/eds/viewarticle?data=dGJyMPPp44rp2%2fdV0%2bnjisfk5Ie46bFMsq6wTbakxmus2%2bKLvq6tSbelsEivqZ5QuKqzUrCmnlnLnPKK3%2bTxeeHq54fs3%2bJVq6euTbOmsFGyq7M%2b6tfsf7vc8D7i2Lt94unjhO6c8nnls79mpNfsVdGnsU2uqrJQtaaxSK6msEi3nOSH8OPfjLvc84Tq6uOQ8gAA&hid=104) Radiation Measurements. 2003 ; 37 : 535–541.

Duller, G.A.T. The Analyst software package for luminescence data: overview and recent improvements. Ancient TL. 2015 ; 33 : 35–42.

Galbraith RF, Roberts RG, Laslett GM, Yoshida H, Olley JM. Optical dating of single and multiple grains of quartz from Jinmium rock shelter, northern Australia: Part I, experimental design and statistical models. Archaeometry. 1999 ; 41(2) : 339-364.

Guibert, P., Lahaye, C., Bechtel, F. The importance of U-series disequilibrium of sediments in luminescence dating: a case study at the Roc de Marsal cave (Dordogne, France). Radiation Measurements. 2009 ; 44(3) : 223-231.

Hansen, V., Murray, A., Buylaert, J.P., Yeo, E.Y.,Thomsen, K., 2015. A new irradiated quartz for beta source calibration. Radiation Measurements, 81, 123-127.

Lebrun, B. Définition du cadre chronologique des gisements préhistoriques de la vallée de la Falémé (Sénégal) et apport des nouvelles techniques de micro-dosimétrie à la datation par luminescence. Thèse de doctorat, Université Bordeaux Montaigne. 2018.

Lebrun, B., Tribolo, C., Chevrier, B., Rasse, M., Lespez, L., Leplongeon, A., Hajdas, I., Abdoulaye, C., Mercier, N., Huysecom, E. West African chrono-cultural framework: first luminescence dating of sedimentary formations from the Falémé Valley, Eastern Senegal. Journal of Archaeological Science Report. 2016 ; 7 : 379-388.

Murray, A.S., Wintle, A.G. Luminescence dating of quartz using an improved single-aliquot regenerative-dose protocol. Radiation Measurements. 2000 ; 32 : 57–73.

Richter, D., Richter, A. and Dornich, K. Lexsyg smart—a luminescence detection system for dosimetry, material research and dating application. Geochronometria. 2015 ; 42(1).

Roberts, R.G., Galbraith, R.F., Yoshida, H., Laslett, G., Olley, J.M. Distinguishing dose populations in sediment mixtures: a test of single-grain optical dating procedures using mixtures of laboratory-dosed quartz. Radiation Measurements. 2000 ; 32 : 459–65.
